# The caspase-2 substrate p54nrb exhibits a multifaceted role in tumor cell death susceptibility via gene regulatory functions

**DOI:** 10.1038/s41419-022-04829-2

**Published:** 2022-04-20

**Authors:** Madeleine Eichler, Ute Distler, Usman Nasrullah, Aswini Krishnan, Manuel Kaulich, Koraljka Husnjak, Wolfgang Eberhardt, Krishnaraj Rajalingam, Stefan Tenzer, Josef Pfeilschifter, Gergely Imre

**Affiliations:** 1 Institute of General Pharmacology and Toxicology, University Hospital Frankfurt, Goethe University Frankfurt, 60590 Frankfurt am Main, Germany; 2 Institute for Immunology, University Medical Center of the Johannes Gutenberg University Mainz, 55131 Mainz, Germany; 3 Present Address: Cell Biology Unit, University Medical Center of the Johannes Gutenberg University Mainz, 55131 Mainz, Germany; 4 Institute of Biochemistry II, Faculty of Medicine, Goethe University Frankfurt, 60590 Frankfurt am Main, Germany; 5 Frankfurt Cancer Institute, 60596 Frankfurt am Main, Germany; 6 Helmholtz-Institute for Translational Oncology Mainz (HI-TRON), Mainz, Germany; 7 German Cancer Research Center (DKFZ), Heidelberg, Germany; 8 Department of Biology and Microbiology, South Dakota State University, Brookings, SD 57007 USA; 9 Department of Molecular Mechanisms of Diseases, University of Zurich-Irchel, CH-8057 Zurich, Switzerland

**Keywords:** Enzyme mechanisms, Apoptosis, Tumour-suppressor proteins

## Abstract

Caspase-2 represents an evolutionary conserved caspase, which plays a role in genotoxic stress-induced apoptosis, ageing-related metabolic changes, and in deleting aneuploid cells in tumors. Genetic deletion of caspase-2 leads to increased tumor susceptibility in vivo. The exact downstream signaling mechanism by which caspase-2 accomplishes its specific tumor suppressor functions is not clear. Caspase-2, uniquely among caspases, resides in the nucleus and other cellular compartments. In this study, we identify a nuclear caspase-2 specific substrate, p54nrb, which is selectively cleaved by caspase-2 at D422, leading to disruption of the C-terminal site, the putative DNA binding region of the protein. P54nrb is an RNA and DNA binding protein, which plays a role in RNA editing, transport, and transcriptional regulation of genes. Overexpression of p54nrb is observed in several human tumor types, such as cervix adenocarcinoma, melanoma, and colon carcinoma. In contrast, the loss of p54nrb in tumor cell lines leads to increased cell death susceptibility and striking decrease in tumorigenic potential. By employing high resolution quantitative proteomics, we demonstrate that the loss/cleavage of p54nrb results in altered expression of oncogenic genes, among which the downregulation of the tumorigenic protease cathepsin-Z and the anti-apoptotic gelsolin can be detected universally across three tumor cell types, including adenocarcinoma, melanoma and colon carcinoma. Finally, we demonstrate that p54nrb interacts with cathepsin-Z and gelsolin DNA, but not RNA. Taken together, this study uncovers a so far not understood mechanism of caspase-2 tumor suppressor function in human tumor cells.

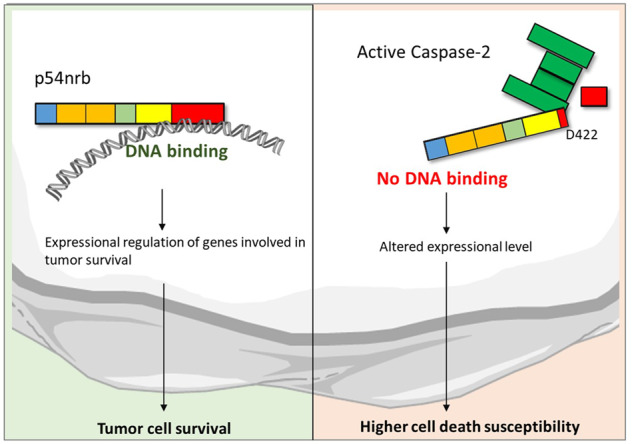

## Introduction

Caspase-2 represents an evolutionary conserved mammalian caspase [1]. Compared to other apoptotic caspases, such as caspase-9, -8, or -3, caspase-2 is less studied due to its pleiotropic and seemingly redundant functions. Caspase-2 is unique among caspases. It exhibits characteristics of initiator caspases such as N-terminal CARD (Caspase Activation and Recruitment Domain) subunit. In addition, caspase-2 also exerts characteristics of executioner caspases and as such can selectively cleave substrates [2, 3] similarly to caspase-3 or -7. Unlike other caspases, caspase-2 has a ubiquitous subcellular localization. For instance, it has been found to reside within the nucleus [4] and nucleolus [5] as well as the Golgi apparatus [6], which implicates its indispensable functions in the processing of compartment-specific substrates. In fact, well supporting these observations, the majority of caspase-2 specific substrates experimentally identified so far reside in subcellular compartments (for instance: Golgin-160 in the Golgi apparatus) [6].

Caspase-2 activation takes place in a protein complex containing p53-Induced Death Domain containing protein (PIDD) and RIPK1 domain containing Adaptor with Death Domain (RAIDD), called the PIDDosome, in response to genotoxic stress [7]. Caspase-2 has also been implicated in ageing-related metabolic changes in mice [8] and has been shown to negatively regulate necroptosis in tumor cells [9]. Alongside caspase-8, caspase-2 is the only caspase that has a demonstrated role in tumor suppression [10, 11]. Its downregulation has been observed in multiple human cancer types [12]. Caspase-2 depletion alone does not lead to carcinogenesis in mouse models, but caspase-2 provides striking protection against oncogene-driven cancers in animal studies [10]. Caspase-2 becomes activated upon cytokinesis failure in a PIDDosome-dependent manner [13] and takes part in the regulation of aneuploidy [14]. These findings provide further evidence for the role of caspase-2 in controlling tumorigenesis. One of the important aspects of caspase-2´s function is that caspase-2 can cleave Mouse Double Minute 2 homolog (MDM-2), the endogenous inhibitor of the tumor suppressor p53 [15]. However, in several human tumors p53 is deleted or mutated, thus non-functional [16]. In addition, p53 driven PIDDosome activation can even facilitate carcinogenesis in hepatocellular carcinoma [17]. These findings rightfully raise the possibility of alternative pathways by which caspase-2 could contribute to tumor suppression. The mechanism by which caspase-2 accomplishes its specific functions, especially in a p53 independent manner, is not characterized [3]. Therefore, our research group set out to identify novel specific substrates of this enigmatic caspase and to decipher their functions.

**Fig. 1:**
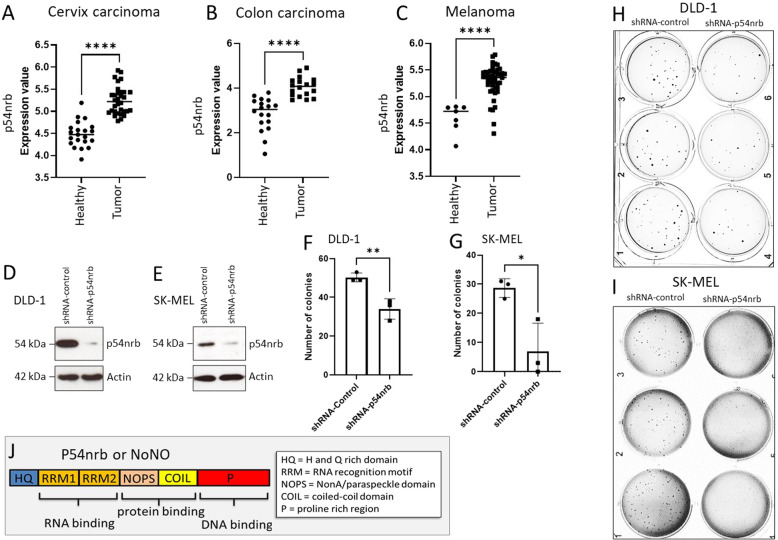
Depletion of p54nrb lowers the viability of tumor cells. P54nrb expression level in healthy tissue versus tumor tissue **A** from cervix carcinoma, **B** from colon carcinoma, and **C** from melanoma patients. Values were obtained from Oncomine database. To test significance Student´s t test was performed. ****p < 0.0001. Immunoblot of p54nrb-level in shRNA-control and shRNA-p54nrb#1 knockdown cells **D** from DLD-1 and **E** from SK-MEL. Counts of colonies of 3D soft agar tumor growth assay of shRNA-control and shRNA-p54nrb#1 cells **F** from DLD-1 and **G** from SK-MEL. 1000 cells/well were seeded and grown for 3 weeks and stained with 0.1% crystal-violet. Student´s t test was performed. *p < 0.05, *n* = 3 (**G**). Representative wells of 3D soft agar tumor growth assay of shRNA-control and shRNA-p54nrb#1 cells **H** from DLD-1 and **I** from SK-MEL cells. **J** Schematic representation of protein domains of p54nrb (also: NoNO): H and Q rich domain (HQ), RNA recognition motif (RRM), NonA/paraspeckle domain (NOPS), coiled-coil domain (COIL), and proline-rich region (P). Brackets indicate the region for RNA, DNA, and protein binding.

P54nrb (nuclear RNA-binding protein 54 kDa) (also: NoNO (Non POU (Pituitary, Octamer transcription factor, Unc) domain-containing octamer-binding protein) is a nuclear RNA and DNA binding protein with transcription regulator activity [18]. P54nrb plays a role in nuclear retention of defective RNAs, RNA-splicing, and transport [19]. P54nrb consists of an N-terminal histidine and glutamine (HQ) rich region, two RNA recognition motifs (RRM), a NOPS domain (C-terminal domain of NONA (protein no-on-transient A), and paraspeckle (PSP) proteins), a charged (+/−) and a proline-rich region (P) at the C-terminal [20] (Fig. 1J). P54nrb was reported to be involved in tumor progression of various tumors, including melanoma [21], and colon carcinoma [22].

In this study, we describe p54nrb as a nuclear caspase-2 substrate and we demonstrate that disruption of p54nrb leads to expressional changes of tumorigenic genes and decreased stress tolerance resulting in increased cell death susceptibility in human tumor cells.

## Results

### Depletion of p54nrb leads to a decrease in key tumorigenic features of tumor cells, including apoptosis tolerance and anchorage-independent growth

**Fig. 2:**
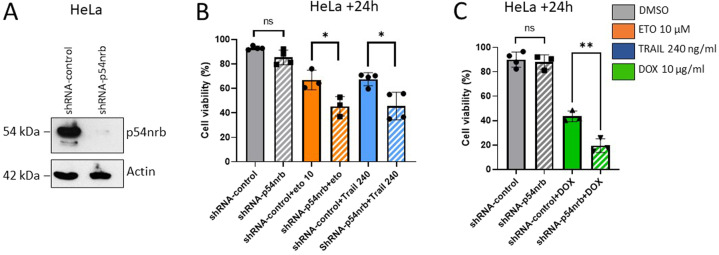
Depletion of p54nrb leads to increased cell death susceptibility. **A** Immunoblot of p54nrb-level in shRNA-control and shRNA-p54nrb#3 knock down HeLa cells. **B** Flow cytometry of HeLa shRNA-control and shRNA-p54nrb#3 cells at 24 h after treatment with 10 µM etoposide (Eto) and 240 ng/ml human recombinant TRAIL (TRAIL). Cell viability was measured by detection and totaling of Annexin-V single positive and Annexin-V and propidium-iodide- double positive cells. The cells not falling to either of these categories were considered as viable cells. Significance was calculated with Student´s t test, *p < 0.05, *n* = 3. **C** Flow cytometry of HeLa shRNA-control and shRNA-p54nrb#3 cells at 24 h after treatment with 10 µg/ml Doxorubicin (DOX). Cell viability was measured by detection and totaling of Annexin-V single positive and Annexin-V and Sytox Blue- double-positive cells. The cells not falling to either of these categories were considered as viable cells. Significance was calculated with Student´s t test, **p < 0.01, *n* = 3.

By employing an online data mining tool (Oncomine.org), we demonstrate that p54nrb expression is upregulated in multiple solid tumor types derived from patient samples, including cervix carcinoma (Fig. 1A) [23], colon carcinoma (Fig. 1B) [24], and melanoma (Fig. 1C) [25]. In order to explore the function of p54nrb in cell death and survival, we employed transient p54nrb gene silencing (Supplementary Fig. 1A) and stable p54nrb knock down in cell lines of the above-described tumor types (Figs. 1D, E and 2A). Unexpectedly, p54nrb knock down alone did not influence the viability of HeLa (human cervix adenocarcinoma), SK-MEL (human melanoma), and DLD-1 (human colon carcinoma) cell lines measured by flow cytometry (Fig. 2B, C and Supplementary Fig. 1A, B, D). In order to assess the long-term survival and colony-forming capacity of p54rnb depleted tumor cell lines, 3D soft agar cell culture assay was employed. In this setting, the knock down of p54nrb significantly reduced the number of tumor colonies formed over a 21-day period in DLD-1 and SK-MEL cells (Fig. 1F-I). In contrast, no significant reduction was observed in HeLa cells (Supplementary Fig. 1C). Therefore, to further evaluate the effect of p54nrb depletion in HeLa cells, we have treated the cells with various apoptosis-inducing chemotherapeutic compounds that stimulate the intrinsic (Etoposide [Eto], Doxorubicin [Dox], and Nocodazole [Noc]) and extrinsic apoptotic pathway (human recombinant TRAIL [TRAIL]). The stable depletion of p54nrb (shRNA-p54nrb) in the HeLa cells led to a significant drop in cell viability compared to that of control cells (shRNA-control) (Fig. 2B, C and Supplementary Fig. 1F, G). This significant cell death sensitization effect was also observed by employing transient p54nrb knock down in Hela cells (Supplementary Fig. 1A). Furthermore, p54nrb knock down SK-MEL and DLD-1 cells with p54nrb knock down also exhibited increased susceptibility to apoptosis induction (Supplementary Fig. 1B, D). The observed cell death sensitization effect was at least partially independent of the function of p53 because the use of a potent p53 inhibitor pifithrin-α did not inhibit the cell death (Supplementary Fig. 1E). Furthermore, overexpression of p54nrb in p54nrb-depleted HeLa cells significantly protected from apoptosis detected by PARP cleavage (Supplementary Fig. 4C, D)

### Depletion and cleavage of p54nrb leads to alteration of expressional patterns of tumor relevant genes, including cathepsin-Z and gelsolin

**Fig. 3:**
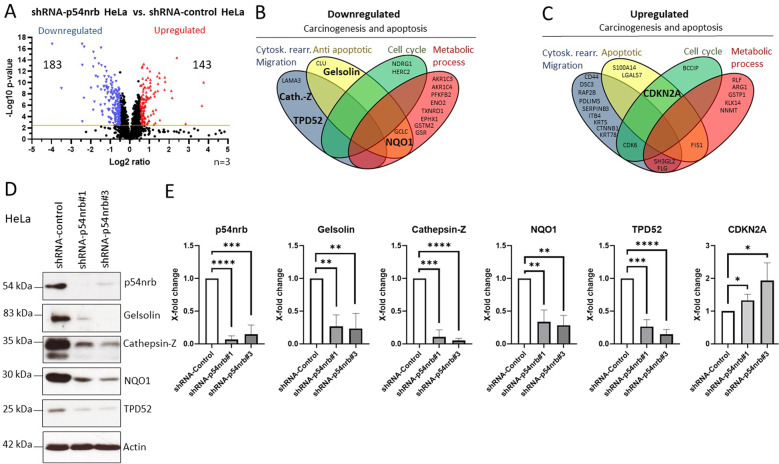
Loss of p54nrb leads to an altered expressional pattern of carcinogenesis relevant genes. **A** Volcano plot diagram of LC/MS data derived from the analysis of HeLa shRNA-control versus shRNA-p54nrb cells. (*n* = 3). Arrowheads depict significantly (*p* < 0.01) upregulated (arrow up, red) and downregulated (arrow down, blue) proteins, and the total numbers of both categories are indicated on the diagram. **B** Up- and **C** downregulated proteins (*p* < 0.01, Log_2_ ratio either >0.5 or < −0.5) of HeLa-shRNA-p54nrb cells compared to shRNA control cells, which exert tumor regulatory and/or apoptosis regulatory properties. Differently colored oval diagrams represent various functional subcategories of these hits. **D** Immunoblot of HeLa shRNA-control and shRNA-p54nrb cells. Detection of p54nrb, gelsolin, cathepsin-Z, NQO1, and TPD52 levels. **E** Quantitative evaluation of immunoblots of p54nrb, gelsolin, cathepsin-Z, NQO1, TPD52, and CDKN2A from HeLa shRNA-control and shRNA-p54nrb#1 and #3 cells. The bands were normalized to the shRNA-control samples. Significance was calculated with one sample t test, *p < 0.05, **p < 0.01, ***p < 0.001, ****p < 0.0001, *n* = 3.

P54nrb is a nuclear protein involved in RNA editing and transcriptional regulation [19]. To analyze the impact of p54nrb depletion on expressional regulation of proteins, we have performed label-free quantitative mass spectrometry. Proteins were considered for further analysis if significant down/upregulation was detected in all three biological replicates (Fig. 3A and Supplementary Fig. 2A). By employing a functional annotation bioinformatics tool (Database for Annotation, Visualization and Integrated Discovery 6.8, Frederick, MD USA) [26], we have selected hits that had experimentally proven relevance in key biological processes involved in cancer progression, such as apoptosis, cytoskeletal rearrangement, migration, cell cycle regulation and metabolic processes (Fig. 3B, C). Next, candidates representing the highest relevance from the four functional cohorts were picked for further validation.

By employing Western blot, cathepsin-Z (cell migration, tumor invasion, proteolysis) [27, 28], gelsolin (apoptosis inhibition) [29], tumor protein D52 (TPD52) (proliferation) [30, 31] and NAD(P)H Quinone Dehydrogenase 1 (NQO1) (metabolic processes) [32] exhibited striking reduction from multiple experiments (Fig. 3D, E), while cyclin-dependent kinase inhibitor 2A (CDKN2A) (cell cycle control) (Fig. 3E and Supplementary Fig. 2B) showed a significant increase in p54nrb knock down HeLa cells. To further substantiate these findings, the protein levels of selected candidates were additionally analyzed in SK-MEL and DLD-1 cells. Intriguingly, TPD52 and NQO1 showed no difference in these tumor cells; however, cathepsin-Z and gelsolin levels were reduced in all p54nrb depleted cell lines (Fig. 3D and Supplementary Fig. 2C, D).

### P54nrb is cleaved in response to apoptosis induction in a caspase-2 dependent manner

**Fig. 4:**
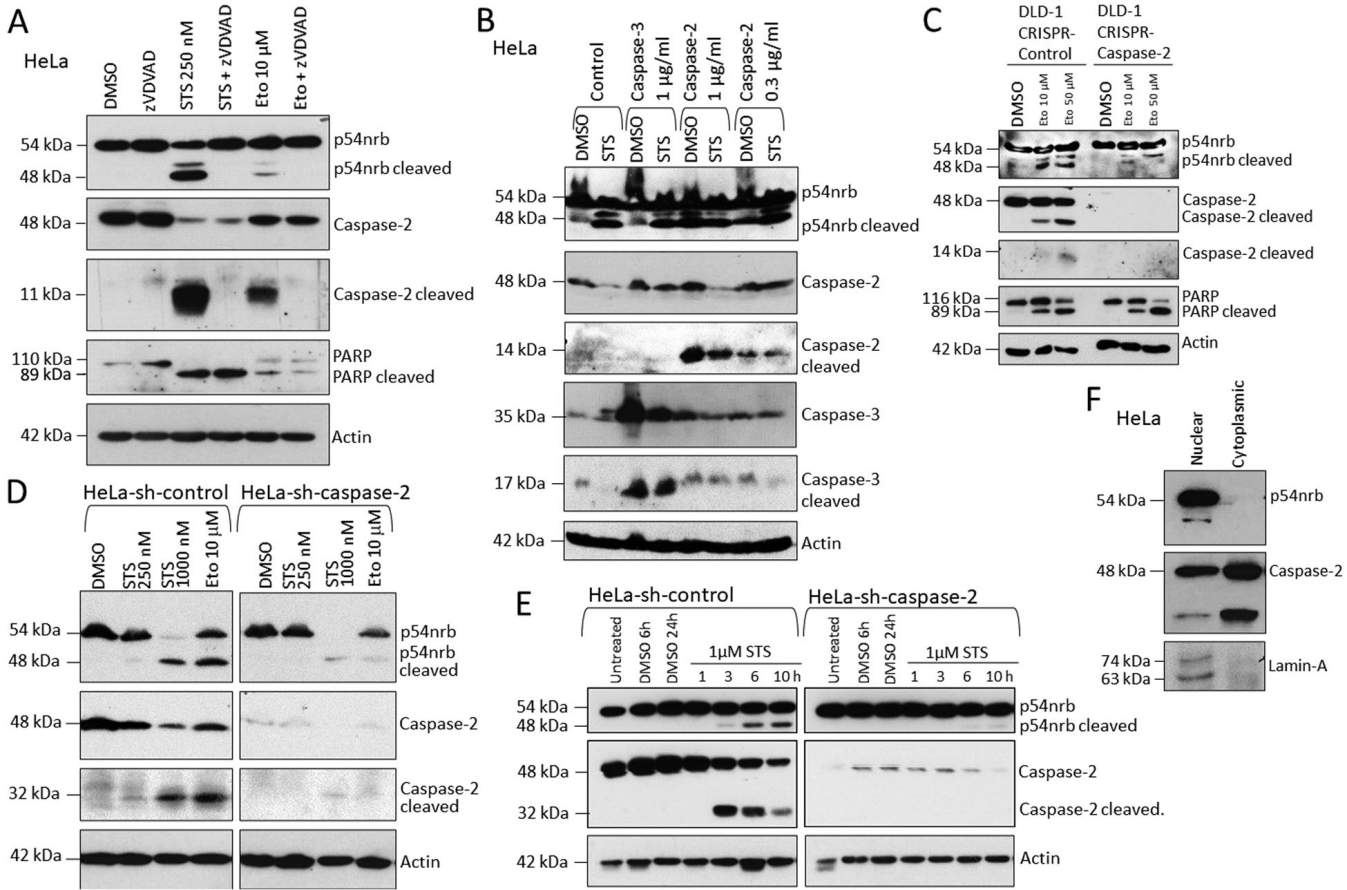
P54nrb is cleaved upon apoptosis stimulation depending on the presence of caspase-2. **A** Immunoblot of p54nrb cleavage in HeLa cells at 24 h after treatment with DMSO or 20 µM Z-VDVAD-fmk (-1 h) and 250 nM staurosporine (STS). **B** Immunoblot of p54nrb cleavage and caspase expression in HeLa cells. HeLa cells were transfected either with 300 or 1000 ng/ml pcDNA3-caspase-2-Flag or pcDNA3-caspase-3-myc, and 24 h later were treated either with DMSO as vehicle control or 250 nM STS. The samples were harvested for Western blot at 24 h. **C** Immunoblot of p54nrb cleavage and caspase-2 in DLD-1 CRISPR-control or DLD-1 CRISPR–caspase-2 cells at 24 h after treatment with DMSO, 10 µM or 50 µM Eto. **D** Immunoblot of p54nrb cleavage and caspase-2 in HeLa shRNA-control or HeLa shRNA-caspase-2 cells at 24 h after treatment with DMSO, 250 nM STS, 1 µM STS or 10 µM Eto. **E** Immunoblot of p54nrb cleavage and caspase-2 in HeLa shRNA-control or HeLa shRNA-caspase-2 cells at 1, 3, 6, and 10 h after treatment with 1 µM STS. DMSO was used as vehicle control. **F** Immunoblot of p54nrb and caspase-2 of nuclear and cytoplasmic fraction of HeLa cells. Lamin-A was detected as nuclear marker.

We identified 2 additional bands of increased electrophoretic mobility (~48 kDa and a weak ~51 kDa fragment) of p54nrb when HeLa cervix adenocarcinoma cells were treated with common apoptosis inducer Staurosporine (STS) (Fig. 4A). The 48 kDa fragment characteristic of caspase-cleaved proteins was also detected upon apoptosis induction in DLD-1 colon carcinoma and SK-MEL melanoma cell lines (Fig. 4C and Supplementary Fig. 3A). Administration of pan-caspase inhibitor z-VAD-fmk (Supplementary Fig. 3D) or caspase-2 inhibitor z-VDVAD-fmk (Fig. 4A) completely blocked the occurrence of the smaller size fragments detected by Western blot. These observations indicated the role of apoptosis and caspases in the production of the lower size fragments of p54nrb. We have previously shown that bacterial pore-forming toxins, such as alpha toxin from *Staphylococcus aureus*, are capable of activating caspase-2 in Hela cells and other epithelial cells [33]. Intriguingly, alpha toxin exposure of Hela cells resulted in the occurrence of the above-observed 48 kDa p54nrb cleavage fragment (Supplementary Fig. 3B) suggesting the role of caspase-2 in the process. A previous study indicated that caspase-3 might cleave p54nrb in vitro [34]. In order to test whether caspase-2 or rather caspase-3 plays a role in p54nrb cleavage in human cells, we have ectopically expressed both caspase-2 and caspase-3 in Hela cells. High local concentration of caspase-2 alone can lead to caspase-2 dimerization and self-cleavage-driven activation [35]. Along with this, increasing concentration of caspase-2 resulted in significant caspase-2 processing and cleavage of p54nrb in a concentration-dependent manner (Fig. 4B). In contrast, caspase-3 or caspase-7 overexpression did not lead to increased p54nrb cleavage (Fig. 4B and Supplementary Fig. 3C). In addition, a pharmacological caspase-3 inhibitor (AQZ-1) did not inhibit the detected cleavage of p54nrb (Supplementary Fig. 3D). To further prove that caspase-2 mediates cleavage of p54nrb, we generated stable CRISPR-Cas-9 caspase-2 depleted (Fig. 4C and Supplementary Fig. 4A) and stable caspase-2 knock down cells (Fig. 4D, E and Supplementary Fig. 4B). The cleavage of p54nrb triggered by apoptosis induction was strikingly diminished in the caspase-2 depleted cells. Caspase-2 is the only caspase that is abundantly present in the nucleus [4]. We have performed subcellular fractionation of the HeLa cells, which identified p54nrb exclusively in the nucleus, and caspase-2 was detectable both in cytoplasm and in nucleus (Fig. 4F). To ensure that caspase-2 and p54nrb co-exist in the nucleus confocal microscopy was performed. This experiment showed that caspase-2 co-localized with p54nrb in the nucleus in dot-like formations (Supplementary Fig. 5A–C). These findings altogether pinpoint the indispensable role of caspase-2 in promoting the cleavage of p54nrb.

The caspase-2 driven cleavage of p54nrb was not altered either by hampering p53 activity (Supplementary Fig. 3F) or in p53 wild type (RKO) and mutated (DLD-1) colon carcinoma cells (Supplementary Fig. 3H and Fig. 4C). This indicates that the cleavage of p54nrb is a p53 independent process.

In addition, we observed that treatment with various apoptosis-inducing compounds resulted in p54nrb cleavage to different extents, and the intensity of p54nrb cleavage/degradation in these samples correlated with the cathepsin-Z protein level (Supplementary Fig. 3E). This is in concordance with the data from p54nrb knock down cells and proves that the cleavage by caspase-2 is the crucial step leading to the downregulation of cathepsin-Z expression.

### Caspase-2 interacts with p54nrb and facilitates the cleavage of p54nrb at D422

**Fig. 5:**
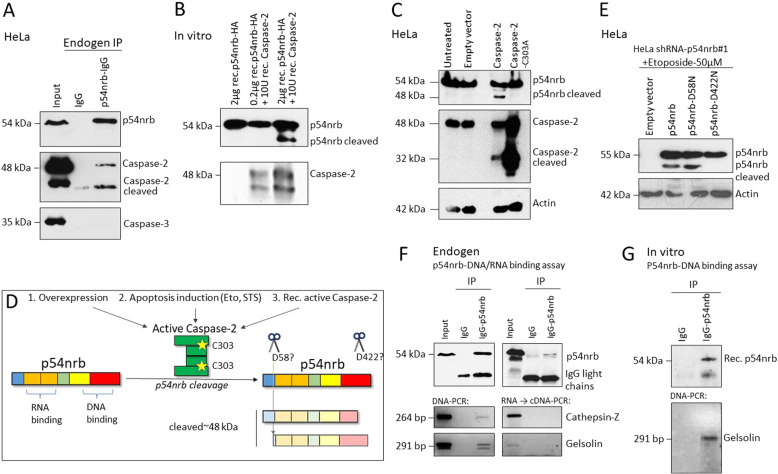
Caspase-2 directly interacts with p54nrb and cleaves p54nrb at D422. **A** Immunoblot of p54nrb caspase-2 and caspase-3 after endogenous immunoprecipitation (IP) of p54nrb from HeLa cells. **B** Immunoblot of p54nrb and caspase-2 after in vitro caspase cleavage assay with 0.2 µg or 2 µg recombinant p54nrb and 10 U human recombinant active caspase-2 after 3 h incubation at 37 °C. **C** Immunoblot of p54nrb cleavage and caspase-2 from HeLa cells at 48 h after ectopic expression of 1 µg/ml empty vector pcDNA3-Flag, pcDNA3-caspase-2-Flag, and pcDNA3-caspase-2-C303A-Flag (inactive). **D** Graphical illustration of p54nrb cleavage by activated caspase-2 via either overexpression, apoptotic induction, or recombinant caspase-2 protein. The active site of caspase-2 dimer is marked with yellow stars. Location of the putative cleavage sites in the p54nrb protein domains are marked with arrows, and its anticipated cleavage fragments are shown. **E** Immunoblot of p54nrb cleavage in HeLa shRNA-p54nrb#1 cells after ectopic expression of pcDNA3-Flag, pFlag-p54nrb, pFlag-p54nrb-D58N and pFlag-p54nrb-D422A (1 µg/ml plasmid) (+24 h) and 24 h treatment with 50 µM Eto. **F** Endogenous ribonucleoprotein immunoprecipitation of p54nrb in HeLa cells and subsequent isolation of coprecipitated DNA or RNA coupled with cDNA-synthesis. Validation of potential co-precipitate by PCR and subsequent agarose gel-electrophoretic detection. **G** In vitro p54nrb/DNA binding assay. 0.5 µg human recombinant p54nrb (Origene, Rockville, MD USA) and 100 ng plasmid encoding the sequence of gelsolin (Ch-gelsoln) [#37262] [Addgene]) were incubated at 37 °C for 18 h. Immunoprecipitation (IP) was performed by employing either p54nrb antibody or the same amount and species of IgG as control. Next, the potentially binding DNA was isolated as described in Methods (see ChIP). P54nrb was detected by western blot and the binding of specific DNA was confirmed by PCR, employing gelsolin-specific primers.

Immunoprecipitation of endogenous p54nrb demonstrated the binding of caspase-2 to p54nrb (Fig. 5A), indicating the direct role of caspase-2 in p54rnb processing. To exclude the role of any intermediate factor between caspase-2´s proteolytic function and p54nrb cleavage, we incubated human recombinant active caspase-2 and human recombinant p54nrb in vitro. Incubation of p54nrb in the presence of active caspase-2 resulted in the occurrence of p54nrb cleaved fragment identical to the 48 kDa cleavage fragment detected in cell lysates (Fig. 5B and Supplementary Fig. 3G). The weaker 51 kDa cleavage fragment occurred specifically upon apoptosis stimulus or caspase-2 activation previously (Fig. 4A–C), however, it was neither detectable in vitro (Fig. 5B), nor consistently detectable in cells. Therefore, we focused on the 48 kDa fragment in the follow-up experiments. Unlike the overexpression of catalytically active caspase-2, the ectopic expression of catalytically inactive (C303A mutation) caspase-2 did not lead to p54nrb cleavage (Fig. 5C), thus proving the direct proteolytic function of caspase-2 in p54nrb cleavage. As caspases in general, caspase-2 also cleaves substrates at aspartate (D) residues [13]. Based on sequence analysis, p54nrb harbors two putative caspase-2 cleavage sites (D58 and D422), cleavage of which is likely to result in the generation of fragments corresponding to 48 kDa (Fig. 5D). To test this hypothesis, we introduced mutations at D58 and D422 of p54nrb and expressed the wild type and mutated proteins in p54nrb depleted cells (Fig. 5E). Treatment with Etoposide (Eto) led to p54nrb cleavage in cells expressing wild type p54nrb and p54nrb-D58N, however, p54nrb-D422N was not prone to cleavage (Fig. 5E). These data together identify p54nrb-D422 as a caspase-2 cleavage site and p54nrb as a novel caspase-2 substrate.

Finally, we set out to investigate the mode of interaction between p54nrb and cathepsin-Z/gelsolin genes. Our previous data (Fig. 5E) demonstrates that the caspase-2 driven cleavage takes place at the putative DNA binding subunit (D422) of p54nrb, suggesting the possible loss of DNA binding capacity. To study the way of interaction between p54nrb and the putative target gene, RNA and Chromatin immunoprecipitations (ChIP) were performed simultaneously by employing a p54nrb specific antibody in Hela cells, and the samples were analyzed by PCR using cathepsin-Z/gelsolin specific primers (Fig. 5F). Interestingly, cathepsin-Z and gelsolin DNA were clearly detectable as the binding partners of p54nrb; however, RNA binding to p54nrb could not be detected in the p54nrb pull down samples. Additionally, the binding between the gene gelsolin and p54nrb was confirmed by an in vitro assay (Fig. 5G). These results highlight the role of p54nrb-DNA interaction in the regulation of gene expression.

**Fig. 6:**
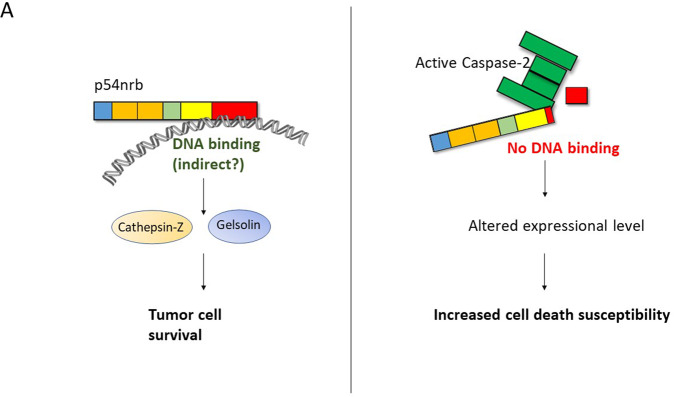
Cleavage by caspase-2 disrupts the gene regulatory function of p54nrb. **A** Schematic illustration of p54nrb’s gene regulatory function in the presence or absence of active caspase-2 and its subsequent effect on tumor cell survival.

Taken together, we identified a novel caspase-2 substrate, p54nrb. P54nrb is a nuclear RNA/DNA binding protein, that is cleaved by caspase-2 at D422, consequently altering the expression of several genes (Fig. 6A). We demonstrated that p54nrb dependent regulation of cathepsin-Z and gelsolin was universal across all the three tumor cell lines investigated. Based on both the location of the cleavage (D422) and the ChIP experiments, we propose a DNA binding dependent regulation for cathepsin-Z and gelsolin in this process (Fig. 6A). Our study provides novel insights into how caspase-2 accomplishes its tumor suppressor function in human carcinoma and melanoma cells, involving p54nrb as a key regulatory factor.

## Discussion

Several in vivo studies underscore the relevance of caspase-2 in tumor suppression. For instance, ATM [36] deficient or constitutive Myc proto-oncogen expressing mice [10] lacking caspase-2 develop lymphoma with significantly higher incidence and rate, whereas Kras expressing mice develop lung cancer with increased incidence when caspase-2 is depleted [37]. Employing a model of mammary tumorigenesis, caspase-2 deficiency leads to higher tumor incidence and decreased survival of mice [38]. How caspase-2 contributes to tumor suppression at the downstream signaling level is still unanswered. This is partly due to the observations that caspase-2 deficiency in several cell types does not lead to cell death inhibition [39]. In contrast, recent studies highlight the relevance of caspase-2 in regulating cell death independent functions in tumor suppression. The most frequently investigated caspase-2 substrate in this context is MDM2, an endogenous inhibitor of p53, which induces cell cycle arrest upon cytokinesis failure. Since this phenomenon is cell and context-specific and many tumors harbor deleted or mutated p53 [40], it is most likely that the anti-tumor function of caspase-2 relies on additional substrates at certain tumor types, such as at the studied cells. For instance, MDM2 overexpression in melanoma is a prognostic marker, which is associated with better clinical outcome [41], thus the cleavage of MDM2 is unlikely to promote tumor suppression there.

We identified p54nrb as a caspase-2 substrate. The depletion of p54nrb did not lead to cell lethality, yet resulted in decrease in long-term tumor cell survival capacity in DLD-1 and SK-MEL cell lines and in increased cell death susceptibility in HeLa, DLD-1, and SK-MEL cells. P54nrb plays a role in expressional regulation of some key tumorigenic genes, identified in this study. Since transcriptional control has a delayed effect on cellular events compared to that of protein level regulation, the activation of caspase-2 bears a more substantial effect on long-term tumor survival than on the outcome of acute cell death in the studied tumor cells. This supports the notion, that the lack of caspase-2 has no significant effect on cell death inhibition [40], but caspase-2 deficient cells tend to proliferate faster [38, 39].

The binding of two tumorigenic genes to p54nrb, cathepsin-Z, and gelsolin have been demonstrated, both of which were significantly downregulated in p54nrb depleted cells confirmed by proteomics and Western blot. Cathepsin-Z is a poorly studied member of the cathepsin family. Apart from its proteolytic function, carcinogenic properties have been also attributed to this protease [27, 28]. Cathepsin-Z interacts with integrins, and promotes their outside-in signaling, leading to focal adhesion complexes and actin cytoskeleton remodeling. Cathepsin-Z promotes tumor cell proliferation via integrins, which aligns well with the decreased tumor cell viability observed in p54nrb depleted cells [42].

Gelsolin acts in actin severing and renders mitochondria to resist mitochondrial depolarization. Consequently, gelsolin overexpression blocks cytochrome-C release from the mitochondria and inhibits the activation of caspases both in vitro and in cells [29]. These findings highlight the importance of caspase-2/p54nrb/gelsolin axis in regulating apoptosis susceptibility.

Along with this, the increased cell death susceptibility of the p54nrb depleted cells was detectable in all three tumor cell lines, meanwhile, the loss of long term tumorigenic capacity was not significant in HeLa cells. The differential expressional regulation between HeLa and the two other cell lines might explain the differences in the tumorigenic capacities in the soft agar cultures. This suggests that the increased cell death susceptibility and the loss of long-term anchorage-independent growth is modulated by distinct p54nrb-regulated factors. In this study, the melanoma cell line responded with the most striking growth inhibition for p54nrb depletion, whereas the colon carcinoma line exhibited moderate effect, and the cervix adenocarcinoma cell line showed no significant changes. The main oncogenic factors of melanoma include the mutations in the retinoblastoma protein signaling [43], which is an important mediator of cell cycle. In this process, the tumor suppressor CDKN2A (also: p16Ink4a) acts as an inhibitor. Our proteomics data showed striking upregulation of CDKN2A in p54nrb depleted cells. However, HeLa cells might not respond to a rising CDKN2A level to a large extent, since mutations in the retinoblastoma gene are not involved in the tumorigenesis of this cervical adenocarcinoma cell line.

In our experiments, we observed two distinct lower molecular weight fragments of p54nrb upon apoptotic stimuli. Next to the well-determined 48 kDa fragment, an approximately 51 kDa fragment occurred specifically upon apoptosis stimulus or caspase-2 activation (Fig. 4A–C). Since this fragment was not detectable in vitro, the role of endogenous post translational modifications or alternative splicing, rather than the second cleavage event is suspected as the main source of the 51 kDa fragment. Moreover, a second putative cleavage site with aspartate residue(s) was not found in the proximity of D422. What type of posttranslational modification can be responsible for the generation of this fragment is not clear. Additionally, the action of a non-aspartase protease can be considered in this process.

Taken together, these data indicate that the studied tumor cells lose their resistance to stressors and become susceptible to cell death induction if the p54nrb level is reduced, whereas the decrease in p54nrb alone does not lead to cell death. This finding is consistent with the data that loss of caspase-2 promotes cancer progression in animal models [10], but caspase-2 depletion itself does not inhibit cell death [3]. Here, we provide evidence that p54nrb as a caspase-2 substrate can play a key role in orchestrating cell death susceptibility and long term tumor survival in distinct tumor types.

## Materials and methods

### Cell culture

HEK-293T (ATCC Manassas, VA USA, CRL-11268), DLD-1 (ATCC, CCL-221), and SK-MEL-28 (ATCC, HTB-72) cells were maintained in Dulbecco’s Modified Eagle Media (DMEM) (Gibco/Thermo Fisher Scientific, Waltham, MA USA), supplemented with 10% fetal bovine serum (FBS), 100 μg/ml streptomycin and 100 units/ml penicillin (both from Merck, Darmstadt, Germany). HeLa cells (ATCC CCL-2) were maintained in Roswell Park Memorial Institute 1640 Media (RPMI) (Gibco), supplemented with 10% FBS, 100 μg/ml streptomycin, and 100 units/ml penicillin. The cells were incubated in cell culture flask at 37 °C with 5% CO_2_. For experiments, cells were cultured in 6- or 12-well cell culture plates. To induce cell death, the following compounds were employed: A-Hemolysin from *Staphylococcus aureus* (α-toxin), Doxorubicin, Etoposide, Staurosporine, Nocodazol and Taxol (Merck, Darmstadt, Germany). In order to inhibit caspase activity, the following compounds were used: AQZ-1/AZ 10417808 (Tocris, Bristol, UK), z-VAD-fmk, and z-VDVAD-fmk (Biozol, Eching, Germany). For inhibition of p53 activity, the following compound was used: Pifithrin-α (Tocris, Bristol, UK).

### Cell transfection with plasmids

Cells were cultured in 6- or 12-well plates until 70–80% confluency. 100 µl/ml OptiMEM (Gibco/Thermo Fisher Scientific, Waltham, MA USA) was mixed with GeneJuice transfection reagent (Ratio of 1 µg DNA: 3 µl GeneJuice) (Merck, Darmstadt, Germany) and incubated at room temperature for 5 min. Then 1 µg plasmid per 1 ml volume of single well was added, mixed, and incubated for further 15 min, dropwise slowly added to the cells and incubated in 5% CO_2_ at 37 °C. The following plasmids were employed: pcDNA3-Flag-HA-1436 (#10792), pcDNA3-caspase-2-Flag (#11811), pcDNA3-caspase-2-C303A-Flag (#11812), pcDNA3-caspase-3-myc (#11813), pcDNA3-caspase-3 C163A-myc (#11814), pcDNA3-caspase-7-Flag (#11815) and Flag-p54nrb (#35379) (all from Addgene, Watertown, MA USA). Additionally, we generated the following plasmids: pCDNA3.1-p54nrb(D58N)-Flag and pCDNA3.1-p54nrb(D422N)-Flag (see also: site-directed mutagenesis).

### Cell transfection with siRNA

Cells were cultured in 12-well plates until 70–80% confluency. 100 µl/ml OptiMEM, 9 µl/ml Hyperfect (Qiagen, Hilden, Germany), and 3 µl siRNA (20 µM siRNA-Control (Qiagen, #1027310) or siRNA-p54nrb#2 (Qiagen, #2999579)) were mixed, incubated for 15 min at room temperature and added dropwise to the cells. Then the cells were incubated in 5% CO_2_ at 37 °C.

### In vitro site-directed mutagenesis

The site-directed mutagenesis was performed on Flag-p54nrb vector. Mutations at the corresponding sequences (D58- > N and D422- > N) were administered by employing the Quickchange II site-directed mutagenesis kit (Agilent, Santa Clara, CA, USA) following the manufacturer´s instructions. The primers harboring the mutated sequences were designed by using the primer design software from Agilent. The mutations in the corresponding sequence were validated by employing standard Sanger sequencing services.

### shRNA lentiviral knock down

For the generation of shRNA lentiviral transduction particles, HEK-293T cells were seeded in six-well plates. At 70–80% confluency the cells were transfected with 4 packaging vectors (each 0.25 µg/ml) and pLenti-shRNA (pLKO.1) (Merck) target-vector (1 µg/ml) using GeneJuice. The supernatant was harvested after 24 h, refilled with medium, and after further 24 h harvested for the second time. The collected supernatants were pooled and filtered using a 0.45 µm pore size filter.

To achieve shRNA knock down 30.000 cells/100 µl were seeded in a 96-well plate. On the next day, the filtered viral supernatants (100 µl/well) or ready-to-use viral particles (Merck) (MOI = 5.6) and 8 µg/ml polybrene (Merck) were added to the cells. After 24 h, the medium was changed and 2.5 µg/ml puromycin (Carl-Roth, Karlsruhe, Germany) was added for selection. After reaching 90–100% confluency, the cells were transferred to a 12-well plate. Next, after reaching 90–100% confluency, the cells were transferred to a six-well plate. Finally, the cells were transferred to a cell culture flask, and samples were taken for knock down validation.

Target sequences of Sigma pLenti shRNA plasmids (Merck):

- ShControl (SHC0161EA): non-target shRNA sequence
- Caspase-2 #4: TRCN0000003508

CCGGGATATGTTGCTCACCACCCTTCTCGAGAAGGGTGGTGAGCAACATATCTTTTT

- p54nrb #1: TRCN0000286693

CCGGGCCAGAATTCTACCCTGGAAACTCGAGTTTCCAGGGTAGAATTCTGGCTTTTTG

- p54nrb #3: TRCN0000074560

CCGGGCAGGCGAAGTCTTCATTCATCTCGAGATGAATGAAGACTTCGCCTGCTTTTTG

### CRSIPR-Cas9 gene editing

To achieve CRISPR-Cas9 directed gene deletion of caspase-2, sgRNA sequences targeting caspase-2 were designed using rule set 2. The sgRNA sequences targeting caspase-2 were cloned into the pLentiCRISPRv2 (Addgene, #98290) by employing BsmBI restriction digestion and ligation based on the protocol described earlier [44].

In order to generate infectious lentiviral particles of pLentiCRISPRv2, HEK-293T cells were seeded in six-well plates to 70–80% confluency and transfected with 1.35 µg/ml pPAX2 (Addgene, #12260) and 0.5 µg/ml pMD2.G (Addgene, #12259) packaging vectors, and a mix of pLentiCRISPRv2 vectors harboring three different sgRNA sequences (1.65 µg/ml) using GeneJuice. The supernatant was harvested after 24 h, refilled with fresh complete medium, and after further 24 h incubation harvested for the second time. The collected supernatants were pooled and filtered using a 0.45 µm poresize filter. To generate CRISPR-Cas9 knockout cells, 30.000 cells/100 µl were seeded in a 96-well plate. On the next day, the filtered viral supernatants (200 µl/well) and 8 µg/ml polybrene were added to the cells. After 24 h, the medium was changed and 2.5 µg/ml puromycin was added for selection. After reaching 90–100% confluency, the cells were transferred to a 12-well plate. Next, after reaching 90–100% confluency, the cells were transferred to a 6-well plate. Finally, the cells were transferred to a cell culture flask, and samples were taken for knock down validation.

Caspase-2 sgRNA Sequences (CACC and AAAC represent BsmBI restriction overhangs):

- No1-Forward: CACCGAGTCACGGACTCCTGCATCG
- No1-Reverse: AAACCGATGCAGGAGTCCGTGACTC
- No2-Forward: CACCGAACTCTAAAAAAGAACCGAG
- No2-Reverse: AAACCTCGGTTCTTTTTTAGAGTTC
- No3-Forward: CACCGAATTCTCACCTGTCGACAGG
- No3-Reverse: AAACCCTGTCGACAGGTGAGAATTC

### Cell harvesting and protein measurement

For harvesting, the cells were detached by use of a cell scraper and centrifuged at 2000 rpm for 4 min. The pellet was washed with 1 ml 5 mM EDTA-PBS and again centrifuged. The pellet was lysed by adding 50 µL lysisbuffer per well of 12-well plate, 80 µl per well of six-well plate, or 100 µl per 10 cm Petri dish respectively. Lysisbuffer was prepared freshly by mixing 1 ml total protein buffer (137 mM NaCl, 20 mM Tris-HCl pH 8, 5 mM EDTA pH 8, 10% glycerol, 1% Triton X-100) with 40 µl 25× Protease-Inhibitor-Mix (Merck), 10 µl Na_3_VO_4_ (*c*_end_ = 1 mM) and 1 µl NaF (*c*_end_ = 1 mM). After resuspension in the lysisbuffer, the cells were frozen five times in liquid nitrogen and thawed at 30 °C in a thermo-block, followed by 30 min of centrifugation at 13.000 rpm at 4 °C. The supernatant was transferred to a new vessel. The protein concentration was determined in duplicates with use of a Pierce BCA protein assay kit (Thermo Fisher Scientific). In a 96-well plate, 1 µl per sample was diluted with 150 µl double-distilled water (dd-H_2_O) and 150 µl working reagent (25 parts of reagent A, 24 parts of reagent B, and 1 part of reagent C) was added. Additionally, a standard curve was established by using BSA in concentrations from 2.5 to 200 µg/ml. The plate was covered and incubated at 60 °C for 45 min. The absorbance was measured at 562 nm on a microplate Elisa-reader using SoftMax Pro software.

### Immunoblot detection

After harvesting the cells and protein measurement, 40 µg protein of lysate was mixed with 4× Laemmli buffer (40% Glycerol, 10% SDS, 125 mM Tris-HCl pH 6.8, 50 mM DTT, 0.01% Bromphenol blue) and dd-H_2_O to the volume of 18 µl. The whole sample was subjected to SDS-PAGE. Subsequently, the samples were transferred to nitrocellulose membrane (0.45 µm, Bio-Rad, Hercules, CA USA) at 70 mA for 80 min. The membrane was blocked for 1 h with 5% milk in TBST (20 mM Tris-HCl pH 7.5, 150 mM NaCl, 0.1% Tween) at room temperature, washed three times for 5 min with TBST, and then incubated with the indicated primary antibody in 5% milk in TBST overnight at 4 °C. Next, the membrane was washed three times for 5 min with TBST, incubated with the corresponding HRP-conjugated secondary antibody for 1 h at room temperature, and washed again three times for 5 min. Finally, the membrane was incubated with ECL solution (Thermo Fisher Scientific) for 5 min and luminescence was detected using a light sensitive X-ray film (GE Healthcare, Chicago, IL USA). The following antibodies were employed: Beta-Actin AC-15 (#A5441) (Merck), Caspase-2 clone 11B4 MAB3507 (Merck) (#11811), Caspase-3 (#9662), Caspase-7 (#9492) (Cell Signaling Technology, Danvers, MA USA), Cathepsin-Z (#ab182575), CDKN2A/p16INK4α EPR1473 (#ab108349) (Abcam, Cambridge, UK), Flag M2-peroxidase (#A8592) (Merck), Gelsolin D9W8Y (#12953) (Cell Signaling Technology), Mouse IgG-HRP Goat (#A0545) (Merck), NQO1 A180 (#39-3700) (Invitrogen, Carlsbad, CA USA), p54nrb/NONO (#A300-587A-1) (Bethyl, Montgomery, TX USA), p54nrb/NONO (#MA32024) (Thermo Fisher Scientific), PARP (#9532) (Cell Signaling Technology), Rabbit IgG-HRP Goat (#A0545) (Merck), Rat IgG-HRP Goat (#01281513) (ENZO, Farmingdale, NY USA) and TPD52 EPR14220 (#ab182578) (Abcam).

### Endogenous immunoprecipitation

Cells were harvested, lysed and the protein concentration was measured as previously described. Then 1 mg of protein lysate was incubated with 30 µl protein-G sepharose beads (GE Healthcare) for 2 h at 4 °C under rotation to pre-clean the lysate of unspecific binding. Subsequently, after a short centrifugation at 2000 rpm for 1 min, the supernatant was transferred into a new vessel and adjusted to 1 ml with lysis buffer. 50 µl of FBS and 1 µg antibody (same amount and species of IgG was used as control) were added and incubated at 4 °C under rotation overnight. Next day, 70 µl protein-G sepharose beads, which were pre-washed 3 times with 500 µl ice-cold lysis buffer and supplemented with protease and phosphatase inhibitor, were added to the overnight samples and incubated for 2 h at 4 °C under rotation. The samples were centrifuged at 10.000 rpm for 1 min at 4 °C and washed as described in the following methods: ChiP and RIP.

### Chromatin Immunoprecipitation (ChIP)

After endogenous precipitation as previously described, the supernatant was removed from the beads that were washed four times for 20 min with 500 µl RIPA buffer (50 mM Tris-HCl pH8, NaCl, 1 % Triton-X100, 0.1% SDS) at 4 °C under rotation. After the last step, the remaining liquid was removed from the beads. Next, the immunoprecipitation was validated by Western blot, and the isolation of DNA was performed simultaneously.

For DNA isolation, the dried beads were resuspended in 200 µl elution buffer A (1% SDS, Tris-EDTA pH 8) and incubated for 10 min at 62 °C and 500 rpm rotation. The samples were centrifuged at 13.000 rpm for 2 min and the supernatant was collected in a new vessel. The beads were now resuspended in 200 µl elution buffer B (0.67% SDS, Tris-EDTA pH 8), incubated at 62 °C and 500 rpm rotation for 10 min, centrifuged, and pooled with the previous. To the pooled supernatant, 365 µl water, 4 µl 5 M NaCl, and 1 µl Rnase A were added and at 65 °C incubated for 4 hours. Subsequently, 2 µl Protein K, 2 µl 0.5 M EDTA pH 8, and 2 µl 1 M Tris-HCl pH 6.5 were added and incubated for 2 hours at 42 °C. Then, samples were mixed with 400 µl basic phenol-chloroform and centrifuged at 13.000 rpm for 15 min at 4 °C. The upper layer was transferred to a new vessel and supplemented with 1 ml ethanol, 40 µl 3 M NaOAc pH 5.2, and 4 µl glycoblue (Thermo Fisher Scientific). DNA was precipitated overnight at −20 °C. The next day, the samples were centrifuged at 13.000 rpm at 4 °C for 15 min and the supernatant was removed. The pellet was washed with 1 ml 70% ice-cold ethanol and centrifuged again. After carefully removing the supernatant, the pellet was dried at 37 °C for 4 min and then resuspended in 20 µl dd-H_2_O. The DNA content was measured with a Nanodrop spectrophotometer (Thermo Fisher Scientific).

### Ribonucleoprotein Immunoprecipitation (RIP)

After endogenous precipitation as previously described, the supernatant was removed from the beads and the beads were washed under rotation three times for 20 min at 4 °C with 500 µl of low salt wash buffer (100 mM NaCl, 50 mM Tris-HCl pH 7.4, 2 mM EDTA pH 8, 2 mM EGTA pH 8, 0.1% SDS, 0.2% TWEEN), followed by three washes with high salt wash buffer (350 mM NaCl, 50 mM Tris-HCl pH 7.4, 2 mM EDTA pH 8, 2 mM EGTA pH 8, 0.1% SDS, 0.2% TWEEN). After the last step, all liquid was removed from the beads. Next, the immunoprecipitation was validated by western blot analysis, and the isolation of RNA was performed simultaneously.

For RNA isolation, 1 ml trizol was added to the dried beads and mixed with 200 µl chloroform for 10 s by vortexing. Samples were centrifuged for 15 min at 13.000 rpm at 4 °C and upper aqueous phase was carefully transferred to a RNase-free vessel. 800 µl isopropanol and 3.5 ml glycoblue were added and incubated over night at −20 °C. Samples were centrifuged at 13.000 rpm for 20 min at 4 °C and the supernatant was carefully removed. The RNA pellet was washed with 500 µl ice-cold 70% ethanol and then dried for 5 min at 37 °C. The pellet was resuspended in 15 µl DEPC-treated water for 10 min at 65 °C. The RNA content was measured with a Nanodrop spectrophotometer (Thermo Fisher Scientific).

### Reverse Transcriptase (RT) reaction

Synthesis of cDNA from RNA via RT reaction was made as follows: 1 µl Random Hexamer Primer, 4 µl 5× RT-PCR reaction buffer, 2 µl 10 mM dNTP-mix (all from Thermo Fisher Scientific) were mixed with 1 µl RNase inhibitor (Biolabs, Cambridge, MA USA) 40 U/µl, 1 µl RevertAid Reverse Transcriptase (Thermo Fisher Scientific) 200 U/µL, 1 µg RNA and adjusted to 20 µl with dd-H_2_O. The sample was incubated at 25 °C for 5 min, followed by 60 min at 37 °C, and terminated with 5 min at 70 °C. The reaction-mix with the newly synthesized cDNA was stored at −20 °C.

### Polymerase Chain Reaction (PCR)

For DNA amplification the following components were mixed per sample: 3 µl 5× Green GoTaq buffer (Promega, Madison, WI USA) 1 µl 50 µM Primer forward, 1 µl 50 µM Primer reverse, 2 µl 10 mM dNTP-mix (Thermo Fisher Scientific), 0.15 µl GoTaq G2 DNA Polymerase (Promega) 5 U/µl, 3 µl 25 mM MgCl_2_, 200 ng DNA-template and adjusted to 25.15 µl with dd-H_2_O. The reaction-mix was incubated at 94 °C for 4 min, followed by 40 cycles of 30 s at 94 °C, 45 s at 55 °C and 45 s at 72 °C, and once a final step for 5 min at 72 °C. PCR amplifications were analyzed by agarose-gel electrophoresis. Therefore, next to 5 µl 100 bp-DNA-ladder, whole sample volumes were loaded onto a 2% (w/v) agarose-TBE gel (TBE buffer: 90 mM Tris-HCl, 80 mM boric acid, 2 mM EDTA pH 8.3) with 5 µl/100 ml midori-green and run at 50 V for 1 h. DNA separation was visualized under a UV transilluminator and documented by using Gel Doc software (Bio-Rad).

Oligonucleotide sequences (Merck):

- Cathepsin-Z Forward: CTCATGTTAAACATTAACCAAG
- Cathepsin-Z Reverse: CTTCCCATCCTTATAGGTG
- Gelsolin Forward: GCCAGTCTAATGAGATATACAC
- Gelsolin Reverse CTTATTTTCACCATTTATCTATATG

### In vitro DNA binding assay

For the in vitro binding assay 0.5 µg human recombinant p54nrb (Origene, Rockville, MD USA) and 100 ng plasmid encoding the sequence of gelsolin (Ch-gelsoln) [#37262] [Addgene]) were adjusted to 20 µl with assay-buffer (0.1 M HEPES, 10% PEG, 0.1% CHAPS, and 10 mM DTT) and incubated at 37 °C for 18 h. 1 ml lysisbuffer, 50 µl of FBS, and 1 µg antibody (or the same amount and species of IgG as control) were added and incubated at 4 °C under rotation overnight. The following steps are equivalent to the protocol of the endogenous immunoprecipitation combined with the ChIP assay.

### Liquid chromatography-mass spectrometry (LC-MS) analysis and label-free quantification analysis

Proteins were extracted and digested using filter-aided sample preparation (FASP) as described in detail before [45, 46]. In brief, samples (corresponding to 20 µg of total protein) were loaded onto spin filter columns (Nanosep centrifugal devices, 30 kDa MWCO; Pall, Port Washington, NY USA). After washing the samples three times with a buffer containing 8 M urea, proteins were reduced and alkylated using dithiothreitol (DDT) and iodoacetamide (IAA), respectively. Excess IAA was quenched by the addition of DTT. Afterwards, the membrane was washed three times with 50 mM NH4HCO_3_ and proteins were digested overnight at 37 °C using trypsin (Trypsin Gold, Promega) at an enzyme-to-protein ratio of 1:50 (w/w). After proteolytic digestion, peptides were recovered by centrifugation. Samples were subsequently acidified with trifluoroacetic acid (TFA) to a final concentration of 1% (v/v) TFA. After lyophilization, purified peptides were reconstituted in 0.1% (v/v) formic acid (FA) for LC-MS analysis.

Tryptic peptides were analyzed as detailed before using a NanoAQUITY UPLC system (Waters Corporation, Milford, MA USA) coupled online to a Synapt G2-S high definition mass spectrometer (Waters Corporation) [46, 47]. In brief, peptides were loaded directly onto an HSS-T3 C18 1.8 μm, 75 μm × 250 mm reversed-phase analytical column (Waters Corporation) running a gradient 5–40% (v/v) mobile phase B (0.1% (v/v) FA and 3% (v/v) DMSO in ACN) at a flow rate of 300 nL/min over 90 min. Mobile phase A was 0.1% (v/v) FA and 3% (v/v) DMSO in water. MS analysis of eluting peptides was performed by ion-mobility separation (IMS) enhanced data-independent acquisition (DIA) in UDMSE mode as described in detail before [46, 47].

The LC-MS raw data were processed in ProteinLynx Global Server (PLGS, ver.3.0.2, Waters Corporation). Data were searched against a custom compiled human proteome database (UniProtKB human reference proteome release 2020) which contained a list of common contaminants. For database search, the following parameters were applied: (i) trypsin as enzyme for digestion, (ii) up to two missed cleavages per peptide, and (iii) peptides had to have a length of at least six amino acids. Carbamidomethyl cysteine was set as fixed, and methionine oxidation as variable modification. The false discovery rate (FDR) for peptide and protein identification was assessed using the target-decoy strategy by searching a reverse database and was set to 0.01 for database search in PLGS.

Postprocessing of data and label-free quantification analysis were performed using the software tool ISOQuant ver.1.8 as detailed before [46, 47]. An FDR of 0.01 at the peptide-level was applied for cluster annotation in ISOQuant. Proteins were only reported if they had been identified by at least two peptides with a minimum length of six amino acids, a minimum PLGS score of 5.5, and no missed cleavages. For each protein, absolute in-sample amounts were calculated using TOP3 quantification [46–48]. To identify significantly regulated proteins (*p* < 0.01), a two tailed t-test was performed and corrected for multiple-hypothesis testing using Benjamini–Hochberg correction. To be included in the final list of significantly regulated proteins, proteins had to be additionally identified in all biological replicates and display a log2-ratio of >0.5 or < −0.5 as compared to the control group.

### Cell death detection by flow cytometry

For cell death analysis, 0.5 × 10^6^ cells were seeded and treated in a 12-well plate. The samples were harvested by collecting the supernatant, washing with 150 µl PBS, incubating for 5 min with 150 µl trypsin-EDTA (Thermo Fisher Scientific), and collecting the detached cells. 300 µl of cell suspension was stained for 30 min with the following solution: 10 µl resuspension buffer (ENZO Lifesciences, Exeter, UK), 0.75 µl 1 M CaCl_2_, 3 µl propidium iodide (PI) (ENZO) or 0.3 µl SytoxBlue (Thermo Fisher Scientific) and 3 µl Annexin-V-EGFP (ENZO). Cell death was measured on FACS Canto-II flow cytometer (BD Bioscience, Franklin Lakes, NJ USA) by using BD FACS Diva software, with employing the PI/FL2 channel (488 nm blue laser/585 nm band‐pass filter) or SytoxBlue/FL1 (405 nm violet laser/450 nm band‐pass filter) and Annexin-V-EGFP/FL1 (488 nm blue laser/530 nm band‐pass filter). Cell debris (population exhibiting low FSC/FL2 intensity) were excluded from the analysis by employing the FSC/FL2 dot-plot.

### Detection of SubG1 population by flow cytometry

For cell death analysis by quantification of the SubG1 population, the cells were harvested for flow cytometry as described previously. Then, the cell suspension was centrifuged at 2000 rpm for 4 min. The pellet was resuspended in 70% ice-cold ethanol for fixation and permeabilization, incubated at room temperature for 15 min, and subsequently stored over night at −20 °C. The samples were centrifuged, and the pellet resuspended in 300 µl extraction buffer (0.2 M Na_2_HPO_4_ pH 7.8), supplemented with 1:1000 RNase-I and incubated for 15 min at room temperature. The samples were then supplemented with 3 µg/ml propidium-iodide (P4864, Sigma-Aldrich) and incubated in dark for 30 min.

The SubG1 population was measured on FACS Canto-II flow cytometer (BD Bioscience, Franklin Lakes, NJ USA) by using BD FACS Diva software. Cell debris (population exhibiting low FSC/FL2 intensity) were excluded from the analysis by employing the FSC/FL2 dot-plot. By employing the PI/FL2 channel (488 nm blue laser/585‐ nm band‐pass filter) the DNA content of each cell was measured, resulting in a cell cycle profile of the sample. The phase below the G1-peak represents the SubG1 population, which has low DNA content due to cell death induced DNA fragmentation.

### 3D soft agar tumor growth assay

Anchorage independent growth was monitored by employing a soft agar assay. First, 1.5 ml/well agar-base (0.75% (w/v) SeaPlaque-Agarose (Lonza, Basel, Switzerland) - DMEM) was poured and solidified in a six-well plate. Then, 1000 cells/well in 1.5 ml agar-topper (0.45% (w/v) SeaPlaque-Agarose - DMEM) were poured and solidified on top of the agar-base. The plate was cultured at 37 °C with 5% CO_2_ for 3 weeks, while pipetting 200 µl DMEM every four days to protect the plate from drying out. For quantitative analysis cell colonies were stained with 0.1% crystal violet (Merck) for 3 h, then washed three times with 1 ml dd-H_2_O for 1 h each. After taking an image of the plate, cell colonies were counted by employing ImageJ.

### In vitro caspase-cleavage assay

For validation of caspase-2 cleavage in vitro, 2 µg human recombinant p54nrb (Origene, Rockville, MD USA) and 10 U human recombinant caspase-2 (ENZO) were mixed and filled up with caspase-buffer (0.1 M HEPES pH 7, 10% PEG, 0.1% CHAPS, 10 mM DTT) to a final volume of 30 µl. The samples were incubated for 3 h at 37 °C. Next, 30 µl of 2× Laemmli was added to the samples and 25 µl of the samples were used for Western blot detection.

### Immunologic staining for fluorescence microscopy

A 8-well µ-slide chamber (Ibidi, Gräfelfing, Germany) was coated with poly L-ysine (10 µg/ml) for 1 h at 37 °C. After washing two times with PBS, 50.000 cells/well were seeded and incubated for 48 h in 5% CO_2_ at 37 °C. The plate was kept on ice, washed two times with PBS and 200 µl ice cold methanol was pipetted on the slides for 4 min and once again washed two times with PBS. The samples were incubated in 5% milk-PBS for 1 h at room temperature. After washing two times with PBS, the primary antibody (2 ng/µl) in 0.1% BSA-PBS was added to the samples for 1 h at room temperature. After two times washing with PBS, the secondary antibody was added (1:1000) in 0.1% BSA-PBS and incubated for 1 h at room temperature in dark, and washed three times in PBS.

The microscopy pictures were taken by employing Laser scanning confocal fluorescence microscopy (LSM 510 Meta) (ZEISS, Jena, Germany) by emplying the ZEN software (ZEISS).

The following antibodies were employed: p54nrb/NONO (#A300-587A-1) (Bethyl, Montgomery, TX USA and caspase-2 (Cell Signaling Technology). Secondary antibodies: anti-rabbit-Alexa-488 and anti-mouse-Alexa-594 (Invitrogen).

### Gene expression analysis in tumor samples

To analyze the expressional level of p54nrb gene in cervical tumors, colon carcinoma, and melanoma, expressional levels were compared to control samples by using Oncomine database (Thermo Fisher Scientific). In the study by Scotto et al RNA isolated from 29 cervical carcinoma cases (20 primary tumors enriched for tumor cells by microdissection and 9 cell lines) and 20 microdisssected normal cervical squamous epithelial cells were used for expression studies. [23]. RNA expressional levels were detected by employing Affymetrix high density microarrays. The array data are available from the NCBI Gene Expression Omnibus using series accession number GSE7803. In the study of Notterman et al gene expression of 18 colon adenocarcinoma and 18 paired normal tissue was measured by using the Human 6500 GeneChip Set (Affymetrix, Santa Clara, CA USA) as previously described [24]. In the study bz Talantov et al the RNA was isolated from 7 normal skin and 45 cutaneous melanoma samples as previously described [25]. The microarray scanning was performed with high-densitiy oligonucleotide microarrays (HG_U133A, Affymetrix).

### Statistical analysis

Sample size (n) is indicated in the figure legends. Statistical significance between two experimental groups was calculated by unpaired Students´t test, two-tailed using GraphPad Prism version 9.1 (GraphPad Software, CA, USA). Center values on graphs are mean values and error bars are standard deviation (sd) of the mean as detailed for each graph in the figure legend.

### Quantitative analysis of Western blots

The quantification of immunoblot detection was performed with imageJ software (Fiji).

## Supplementary information

authors confirmation on author addition

Suppl Fig1

Suppl Fig2

Suppl Fig3

Suppl Fig4

Suppl Fig5

Supplementary Fig legends

aj-checklist

Author contribution form

## Data Availability

The datasets used and analyzed during the current study are available from the corresponding author on reasonable request.

## References

[1] Kumar S, Kinoshita M, Noda M, Copeland NG, Jenkins NA (1994). Induction of apoptosis by the mouse Nedd2 gene, which encodes a protein similar to the product of the Caenorhabditis elegans cell death gene ced-3 and the mammalian IL-1 beta-converting enzyme. Genes Dev.

[2] Wejda M, Impens F, Takahashi N, Van Damme P, Gevaert K, Vandenabeele P (2012). Degradomics reveals that cleavage specificity profiles of caspase-2 and effector caspases are alike. J Biol Chem.

[3] Brown-Suedel AN, Bouchier-Hayes L (2020). Caspase-2 substrates: To apoptosis, cell cycle control, and beyond. Front Cell Developmental Biol.

[4] Janssens S, Tinel A (2012). The PIDDosome, DNA-damage-induced apoptosis, and beyond. Cell Death Differ.

[5] Ando K, Parsons MJ, Shah RB, Charendoff CI, Paris SL, Liu PH (2017). NPM1 directs PIDDosome-dependent caspase-2 activation in the nucleolus. J Cell Biol.

[6] Mancini M, Machamer CE, Roy S, Nicholson DW, Thornberry NA, Casciola-Rosen LA (2000). Caspase-2 is localized at the Golgi complex and cleaves golgin-160 during apoptosis. J Cell Biol.

[7] Tinel A, Tschopp J (2004). The PIDDosome, a protein complex implicated in activation of caspase-2 in response to genotoxic stress. Science..

[8] Wilson CH, Shalini S, Filipovska A, Richman TR, Davies S, Martin SD (2015). Age-related proteostasis and metabolic alterations in Caspase-2-deficient mice. Cell Death Dis.

[9] Zamaraev AV, Kopeina GS, Buchbinder JH, Zhivotovsky B, Lavrik IN (2015). Caspase-2 is a negative regulator of necroptosis. Int J Biochem Cell Biol.

[10] Ho LH, Taylor R, Dorstyn L, Cakouros D, Bouillet P, Kumar S (2009). A tumor suppressor function for caspase-2. Proc Natl Acad Sci USA.

[11] Kopeina GS, Zhivotovsky B (2021). Caspase-2 as a master regulator of genomic stability. Trends cell Biol.

[12] Ren K, Lu J, Porollo A, Du C (2012). Tumor-suppressing function of caspase-2 requires catalytic site Cys-320 and site Ser-139 in mice. J Biol Chem.

[13] Fava LL, Bock FJ, Geley S, Villunger A (2012). Caspase-2 at a glance. J Cell Sci.

[14] Dawar S, Lim Y, Puccini J, White M, Thomas P, Bouchier-Hayes L (2017). Caspase-2-mediated cell death is required for deleting aneuploid cells. Oncogene..

[15] Lim Y, Dorstyn L, Kumar S (2021). The p53-caspase-2 axis in the cell cycle and DNA damage response. Exp Mol Med.

[16] Muller PA, Vousde KH (2013). p53 mutations in cancer. Nat Cell Biol.

[17] Sladky VC, Knapp K, Szabo TG, Braun VZ, Bongiovanni L, van den Bos H (2020). PIDDosome-induced p53-dependent ploidy restriction facilitates hepatocarcinogenesis. EMBO Rep.

[18] Dong B, Horowitz DS, Kobayashi R, Krainer AR (1993). Purification and cDNA cloning of HeLa cell p54nrb, a nuclear protein with two RNA recognition motifs and extensive homology to human splicing factor PSF and Drosophila NONA/BJ6. Nucleic Acids Res.

[19] Shav-Tal Y, Zipori D (2002). PSF and p54(nrb)/NonO—multi-functional nuclear proteins. FEBS Lett.

[20] Duvignaud JB, Bédard M, Nagata T, Muto Y, Yokoyama S, Gagné SM (2016). Structure, dynamics, and interaction of p54(nrb)/NonO RRM1 with 5’ splice site RNA sequence. Biochemistry..

[21] Schiffner S, Zimara N, Schmid R, Bosserhoff AK (2011). p54nrb is a new regulator of progression of malignant melanoma. Carcinogenesis.

[22] Nelson LD, Bender C, Mannsperger H, Buergy D, Kambakamba P, Mudduluru G (2012). Triplex DNA-binding proteins are associated with clinical outcomes revealed by proteomic measurements in patients with colorectal cancer. Mol Cancer.

[23] Scotto L, Narayan G, Nandula SV, Arias-Pulido H, Subramaniyam S, Schneider A (2008). Identification of copy number gain and overexpressed genes on chromosome arm 20q by an integrative genomic approach in cervical cancer: potential role in progression. Genes Chromosomes Cancer.

[24] Notterman DA, Alon U, Sierk AJ, Levine AJ (2001). Transcriptional gene expression profiles of colorectal adenoma, adenocarcinoma, and normal tissue examined by oligonucleotide array. Cancer Res.

[25] Talantov D, Mazumder A, Yu JX, Briggs T, Jiang Y, Backus J (2005). Novel genes associated with malignant melanoma but not benign melanocytic lesions. Clin Cancer Res.

[26] Huang D, Sherman BT, Lempicki RA (2009). Systematic and integrative analysis of large gene lists using DAVID bioinformatics resources. Nat Protoc.

[27] Wang J, Chen L, Li Y, Guan XY (2011). Overexpression of cathepsin Z contributes to tumor metastasis by inducing epithelial-mesenchymal transition in hepatocellular carcinoma. PloS One.

[28] Mitrović A, Pečar Fonović U, Kos J (2017). Cysteine cathepsins B and X promote epithelial-mesenchymal transition of tumor cells. Eur J Cell Biol.

[29] Koya RC, Fujita H, Shimizu S, Ohtsu M, Takimoto M, Tsujimoto Y (2000). Gelsolin inhibits apoptosis by blocking mitochondrial membrane potential loss and cytochrome c release. J Biol Chem.

[30] Ummanni R, Teller S, Junker H, Zimmermann U, Ven S, Scharf C (2008). Altered expression of tumor protein D52 regulates apoptosis and migration of prostate cancer cells. FEBS J.

[31] Wang Z, Li Y, Fan L, Zhao Q, Tan B, Liu R (2020). Silencing of TPD52 inhibits proliferation, migration, invasion but induces apoptosis of pancreatic cancer cells by deactivating Akt pathway. Neoplasma.

[32] Zhou HZ, Zeng HQ, YuanD, Ren JH, Cheng ST, Yu HB (2019). NQO1 potentiates apoptosis evasion and upregulates XIAP via inhibiting proteasome-mediated degradation SIRT6 in hepatocellular carcinoma. Cell Commun Signal.

[33] Imre G, Heering J, Takeda AN, Husmann M, Thiede B, zu Heringdorf DM (2012). Caspase-2 is an initiator caspase responsible for pore-forming toxin-mediated apoptosis. EMBO J.

[34] Thiede B, Dimmler C, Siejak F, Rudel T (2001). Predominant identification of RNA-binding proteins in Fas-induced apoptosis by proteome analysis. J Biol Chem.

[35] Butt AJ, Harvey NL, Parasivam G, Kumar S (1998). Dimerization and autoprocessing of the Nedd2 (caspase-2) precursor requires both the prodomain and the carboxyl-terminal regions. J Biol Chem.

[36] Puccini J, Shalini S, Voss AK, Gatei M, Wilson CH, Hiwase DK (2013). Loss of caspase-2 augments lymphomagenesis and enhances genomic instability in Atm-deficient mice. Proc Natl Acad Sci USA..

[37] Terry MR, Arya R, Mukhopadhyay A, Berrett KC, Clair PM, Witt B (2015). Caspase-2 impacts lung tumorigenesis and chemotherapy response in vivo. Cell Death Differ.

[38] Parsons MJ, McCormick L, Janke L, Howard A, Bouchier-Hayes L, Green DR (2013). Genetic deletion of caspase-2 accelerates MMTV/c-neu-driven mammary carcinogenesis in mice. Cell Death Differ.

[39] Bouchier-Hayes L, Green DR (2012). Caspase-2: The orphan caspase. Cell Death Differ.

[40] Sidi S, Sanda T, Kennedy RD, Hagen AT, Jette CA, Hoffmans R (2008). Chk1 suppresses a caspase-2 apoptotic response to DNA damage that bypasses p53, Bcl-2, and caspase-3. Cell.

[41] Polsky D, Melzer K, Hazan C, Panageas KS, Busam K, Drobnjak M (2002). HDM2 protein overexpression and prognosis in primary malignant melanoma. J Natl Cancer Inst.

[42] Akkari L, Gocheva V, Keste JC, Hunter KE, Quick ML, Sevenich L (2014). Distinct functions of macrophage-derived and cancer cell-derived cathepsin Z combine to promote tumor malignancy via interactions with the extracellular matrix. Genes Dev.

[43] Polsky D, Cordon-Cardo C (2003). Oncogenes in melanoma. Oncogene.

[44] Wegner M, Diehl V, Bittl V, de Bruyn R, Wiechmann S, Matthess Y (2009). Circular synthesized CRISPR/Cas gRNAs for functional interrogations in the coding and noncoding genome. eLife..

[45] Wisniewski JR, Zougman A, Nagaraj N, Mann M (2009). Universal sample preparation method for proteome analysis. Nat Methods.

[46] Distler U, Kuharev J, Navarro P, Tenzer S (2016). Label-free quantification in ion mobility-enhanced data-independent acquisition proteomics. Nat Protoc.

[47] Distler U, Kuharev J, Navarro P, Levin Y, Schild H, Tenzer S (2014). Drift time-specific collision energies enable deep-coverage data-independent acquisition proteomics. Nat Methods.

[48] Silva JC, Gorenstein MV, Li GZ, Vissers JPC, Geromanos SJ (2006). Absolute quantification of proteins by LCMSE: A virtue of parallel MS acquisition. Mol Cell Proteom.

