# Supplementary material for: The caspase-2 substrate p54nrb exhibits a multifaceted role in tumor cell death susceptibility via gene regulatory functions
Source: Cell Death Dis. 2022 Apr 20;13(4):386. doi: 10.1038/s41419-022-04829-2 (PMC9021192; doi:10.1038/s41419-022-04829-2)
Supplement: Supplementary file 1 — authors confirmation on author addition [file 41419_2022_4829_MOESM1_ESM.pdf]

Re: confirmation of changes requested by the journal - Message (HTML)

File Message Help Tell me what you want to do

Re: confirmation of changes requested by the journal - Message (HTML)

File Message Help Tell me what you want to do

Ignore Junk Delete Archive | Reply Reply All Forward Meeting More | Move to: ? Team Email Create New To Manager Reply & Delete | Move Rules Actions | Mark Unread Follow Up | Find Related Select | Read Aloud Immersive Reader | Translate | Zoom

Delete Respond Quick Steps Move Tags Editing Immersive Language Zoom

Re: confirmation of changes requested by the journal

KH Koraljka Husnjak <k.husnjak@biochem2.uni-frankfurt.de>
To gimre80@gmail.com

Reply Reply All Forward

Mi 30.03.2022 11:19

Dear Gergely,

I agree to the addition of an author to the manuscript.

Congratulations for your manuscript and best regards,
Koraljka

Dr. Koraljka Husnjak

Ubiquitin Signaling Group
Frankfurt CRISPR/Cas Screening Center
Institute of Biochemistry II
Gustav Embden-Zentrum der Biochemie
Goethe University Frankfurt – Medical Faculty
University Hospital

Theodor-Stern-Kai 7, Building 75
60590 Frankfurt, Germany
Tel: +49 69 6301 5820
Fax: +49 69 6301 7603
K.Husnjak@biochem2.uni-frankfurt.de
www.biochem2.com

3°C Bewölkt DEU 13:01 02.04.2022

Re: confirmation of changes requested by the journal - Message (HTML)

File Message Help Tell me what you want to do

Ignore Junk Delete Archive | Delete
Reply Reply All Forward Meeting More | Respond
Move to: ? Team Email Create New To Manager Reply & Delete | Quick Steps
Move Rules Actions | Move
Mark Unread Follow Up | Tags
Find Related Select | Editing
Read Aloud Immersive Reader | Immersive
Translate | Language
Zoom | Zoom

Re: confirmation of changes requested by the journal

TS Tenzer, Stefan <tenzer@uni-mainz.de>
To Gergely Imre
Cc Krishna, Prof. Rajalingam; Madeleine Eichler; Distler, Ute; Manuel Kaulich; Koraljka Husnjak; Usman Nasrullah
Signed By There are problems with the signature. Click the signature button for details.

Reply Reply All Forward

Do 31.03.2022 01:55

Dear Greg,

I fully agree with the addition of an author to the manuscript.

Best regards,

Stefan

Univ.-Prof. Dr. rer. nat. Stefan Tenzer

HI-TRON Mainz Brückenprofessur Immunoproteomik

UNIVERSITÄTSMEDIZIN
der Johannes Gutenberg-Universität Mainz
Institut für Immunologie
Core Facility für Massenspektrometrie
www.immunologie-mainz.de

Helmholtz Institute for Translational Oncology Mainz (HI-TRON Mainz)
A Helmholtz Institute by DKFZ

Deutsches Krebsforschungszentrum (DKFZ)
Stiftung des öffentlichen Rechts

Re: confirmation of changes requested by the journal - Message (Plain Text)

File Message Help Tell me what you want to do

Re: confirmation of changes requested by the journal

Pfeilschifter@em.uni-frankfurt.de
To gimre80@gmail.com

Fr 01.04.2022 04:49

We removed extra line breaks from this message.

Dear Greg,

I agree to the addition of an author to the manuscript.
Best regards,

Josef Pfeilschifter

Zitat von gimre80@gmail.com:

> Dear co-aouthors,
>
>
>
> As you might know, the paper has been provisionally accepted in Cell
> Death an Disease.
>
> Now, the journal would like to receive the confirmation from all
> authors regarding the changes in the author list. You have already
> seen this version of the manuscript, however now please send me a
> reply e-mail with a confirmation ("I agree to the addition of an
> author to the manuscript") at your earliest convenience. The last
> version of the manuscript has been attached. Also, if any changes in

Re: confirmation of changes requested by the journal - Message (HTML)

File Message Help Tell me what you want to do

Ignore Junk Delete Archive Reply Reply All Forward Meeting More Move to: ? Team Email Create New To Manager Reply & Delete Move Rules Actions Mark Unread Follow Up Find Related Select Read Aloud Immersive Reader Translate Zoom

Delete Respond Quick Steps Move Tags Editing Immersive Language Zoom

Re: confirmation of changes requested by the journal

KP Krishna, Prof. Rajalingam <krishna@uni-mainz.de>
To gimre80@gmail.com

Reply Reply All Forward

Mi 30.03.2022 12:4

Dear Greg,

I agree to the addition of an author to the manuscript

Best
Krishna

From: Gergely Imre <gimre80@gmail.com>
Date: Wednesday, 30. March 2022 at 17:00
To: "Tenzer, Stefan" <tenzer@uni-mainz.de>, "Krishna, Prof. Rajalingam" <krishna@uni-mainz.de>, Joseph Pfeilschifter <Pfeilschifter@em.uni-frankfurt.de>, 'Madeleine Eichler' <eichler.madeleine@gmail.com>, 'Usman Nasrullah' <usman.nasrullah111@yahoo.com>, "Distler, Ute" <ute.distler@uni-mainz.de>, Manuel Kaulich <kaulich@em.uni-frankfurt.de>, 'Koraljka Husnjak' <k.husnjak@biochem2.uni-frankfurt.de>, Aswini Krishnan <krishnan.aswini@gmail.com>, Wolfgang Eberhardt <w.eberhardt@em.uni-frankfurt.de>
Cc: "gergely.imre@sdstate.edu" <gergely.imre@sdstate.edu>
Subject: confirmation of changes requested by the journal

Dear co-aouthors,

As you might know, the paper has been provisionally accepted in Cell Death an Disease.
Now, the journal would like to receive the confirmation from all authors regarding the changes in the author list. You have already seen this version of the manuscript, however now please send me a reply e-mail with a confirmation ("I agree to the addition of an author to the manuscript") at your earliest convenience. The last version of the manuscript has been attached. Also, if any changes in affiliation etc need to be made, please let mem know.

Thank you for your cooperation,

Re: confirmation of changes requested by the journal - Message (HTML)

File Message Help Tell me what you want to do

Ignore Junk Delete Archive | Delete
Reply Reply All Forward Meeting More | Respond
Move to: ? Team Email Create New To Manager Reply & Delete | Quick Steps
Move Rules Actions | Move
Mark Unread Follow Up | Tags
Find Related Select | Editing
Read Aloud Immersive Reader | Immersive
Translate | Language
Zoom | Zoom

Re: confirmation of changes requested by the journal

UN Usman Nasrullah <usman.nasrullah111@yahoo.com>
To gimre80@gmail.com; 'Tenzer, Stefan'; 'Krishna, Prof. Rajalingam'; 'Madeleine Eichler'; 'Distler, Ute'; 'Manuel Kaulich'; 'Koraljka Husnjak'

Reply Reply All Forward

Mi 30.03.2022 12:44

Dear Dr. Imre,
I totally agree with the addition of an author to the manuscript.
Thank you
Kind regards,
Usman Nasrullah

Sent from Yahoo Mail on Android

> Dear co-aouthors,
>
> As you might know, the paper has been provisionally accepted in Cell Death an Disease.
>
> Now, the journal would like to receive the confirmation from all authors regarding the changes in the author list. You have already seen this version of the manuscript, however now please send me a reply e-mail with a confirmation ("I agree to the addition of an author to the manuscript") at your earliest convenience. The last version of the manuscript has been attached. Also, if any changes in affiliation etc need to be made, please let mem know.
>
> Thank you for your cooperation,

Re: confirmation of changes requested by the journal - Message (HTML)

File Message Help Tell me what you want to do

Ignore Junk Delete Archive Reply Reply All Forward Meeting More Move to: ? Team Email Create New To Manager Reply & Delete Move Rules Actions Mark Unread Follow Up Find Related Select Read Aloud Immersive Reader Translate Zoom

Delete Respond Quick Steps Move Tags Editing Immersive Language Zoom

Re: confirmation of changes requested by the journal

DU Distler, Ute <ute.distler@uni-mainz.de>

To gimre80@gmail.com

Reply

Dear Greg,

These are good news :-)

Regarding the authorship:
I agree to the addition of an author to the manuscript.

Do you also need an official signature?

Best regards,

Ute

PS.: Looking at the latest version of the manuscript I realized that the affiliations are not in the right order (e.g. 3 before 2), but most likely you are already aware of it.

PD Dr. Ute Distler
Institute for Immunology
____
Core Facility for Mass Spectrometry

Re: confirmation of changes requested by the journal - Message (HTML)

File Message Help Tell me what you want to do

Ignore Delete Archive Reply Reply All Forward Meeting More Move to: ? To Manager Team Email Reply & Delete Create New Move Rules Actions Mark Unread Follow Up Find Related Select Read Aloud Immersive Reader Translate Zoom

Delete Respond Quick Steps Move Tags Editing Immersive Language Zoom

Re: confirmation of changes requested by the journal

MK Manuel Kaulich <kaulich@em.uni-frankfurt.de>

To gimre80@gmail.com; gergely.imre@sdstate.edu

Reply Reply All Forward ...

Mi 30.03.2022 10:16

Manuscript text Eichler et al_03-26-22_Main Text_MK.docx
94 KB

Untitled attachment 00052.html
4 KB

Dear Greg,

I agree to the addition of an author to the manuscript.

Please note that I added the FCI affiliation to my name, attached.

Best wishes
Manuel

Manuel Kaulich, PhD
Group Leader - Functional Genomics & Genetic Vulnerabilities

University Hospital of Goethe University Frankfurt
Building 75, Ground floor, Room 0.222
Theodor Stern Kai 7
60590 Frankfurt am Main, Germany

Kaulich Lab
T +49 69 6301 5450
Twitter @ManuelKaulich

Re: confirmation of changes requested by the journal - Message (HTML)

File Message Help Tell me what you want to do

Re: confirmation of changes requested by the journal - Message (HTML)

File Message Help Tell me what you want to do

Ignore Junk Delete Archive | Reply Reply All Forward Meeting More | Move to: ? Team Email Create New To Manager Reply & Delete | Move Rules Actions | Mark Unread Follow Up | Find Related Select | Read Aloud Immersive Reader | Translate | Zoom

Delete Respond Quick Steps Move Tags Editing Immersive Language Zoom

Re: confirmation of changes requested by the journal

KH Koraljka Husnjak <k.husnjak@biochem2.uni-frankfurt.de>

To gimre80@gmail.com

Reply Reply All Forward ...

Mi 30.03.2022 11:19

Dear Gergely,

I agree to the addition of an author to the manuscript.

Congratulations for your manuscript and best regards,
Koraljka

Dr. Koraljka Husnjak

Ubiquitin Signaling Group
Frankfurt CRISPR/Cas Screening Center
Institute of Biochemistry II
Gustav Embden-Zentrum der Biochemie
Goethe University Frankfurt – Medical Faculty
University Hospital

Theodor-Stern-Kai 7, Building 75
60590 Frankfurt, Germany
Tel: +49 69 6301 5820
Fax: +49 69 6301 7603
K.Husnjak@biochem2.uni-frankfurt.de
www.biochem2.com

Thank you for your cooperation,

Re: confirmation of changes requested by the journal - Message (HTML)

File | Message | Help | Tell me what you want to do

Ignore | Junk | Delete | Archive | Reply | Reply All | Forward | Meeting | More | Move to: ? | Team Email | Create New | To Manager | Reply & Delete | Move | Rules | Actions | Mark Unread | Follow Up | Find | Related | Select | Read Aloud | Immersive Reader | Translate | Zoom

Delete | Respond | Quick Steps | Move | Tags | Editing | Immersive | Language | Zoom

Re: confirmation of changes requested by the journal

KH Koraljka Husnjak <k.husnjak@biochem2.uni-frankfurt.de>
To gimre80@gmail.com

Reply | Reply All | Forward

Mi 30.03.2022 11:19

Dear Gergely,

I agree to the addition of an author to the manuscript.

Congratulations for your manuscript and best regards,
Koraljka

Dr. Koraljka Husnjak

Ubiquitin Signaling Group
Frankfurt CRISPR/Cas Screening Center
Institute of Biochemistry II
Gustav Embden-Zentrum der Biochemie
Goethe University Frankfurt – Medical Faculty
University Hospital

Theodor-Stern-Kai 7, Building 75
60590 Frankfurt, Germany
Tel: +49 69 6301 5820
Fax: +49 69 6301 7603
K.Husnjak@biochem2.uni-frankfurt.de
www.biochem2.com

Re: confirmation of changes requested by the journal - Message (HTML)

File Message Help Tell me what you want to do

Ignore Junk Delete Archive Reply Reply All Forward Meeting More Move to: ? Team Email Create New To Manager Reply & Delete Move Rules Actions Mark Unread Follow Up Find Related Select Read Aloud Immersive Reader Translate Zoom

Delete Respond Quick Steps Move Tags Editing Immersive Language Zoom

Re: confirmation of changes requested by the journal

WF w.eberhardt@em.uni-frankfurt.de
To gimre80@gmail.com

Reply Reply All Forward

Do 31.03.2022 02:59

Dear Greg,

here is my official confirmation to "Cell Death and Diseases":

To whom it may concern:

I agree to the addition of an author to the manuscript to the manuscript entitled

"The caspase-2 substrate p54nrb exhibits a multifaceted role in tumor cell death susceptibility via gene regulatory functions"

by Madeleine Eichler, Ute Distler, Usman Nasrullah, Aswini Krishnan, Manuel Kaulich, Koraljka Husnjak, Wolfgang Eberhardt, Krishnaraj Rajalingam, Stefan Tenzer, Josef Pfeilschifter, Gergely Imre.

Kind regards,

Wolfgang Eberhardt, PhD
