# Supplementary figures and images for: The caspase-2 substrate p54nrb exhibits a multifaceted role in tumor cell death susceptibility via gene regulatory functions
Source: Cell Death Dis. 2022 Apr 20;13(4):386. doi: 10.1038/s41419-022-04829-2 (PMC9021192; doi:10.1038/s41419-022-04829-2)

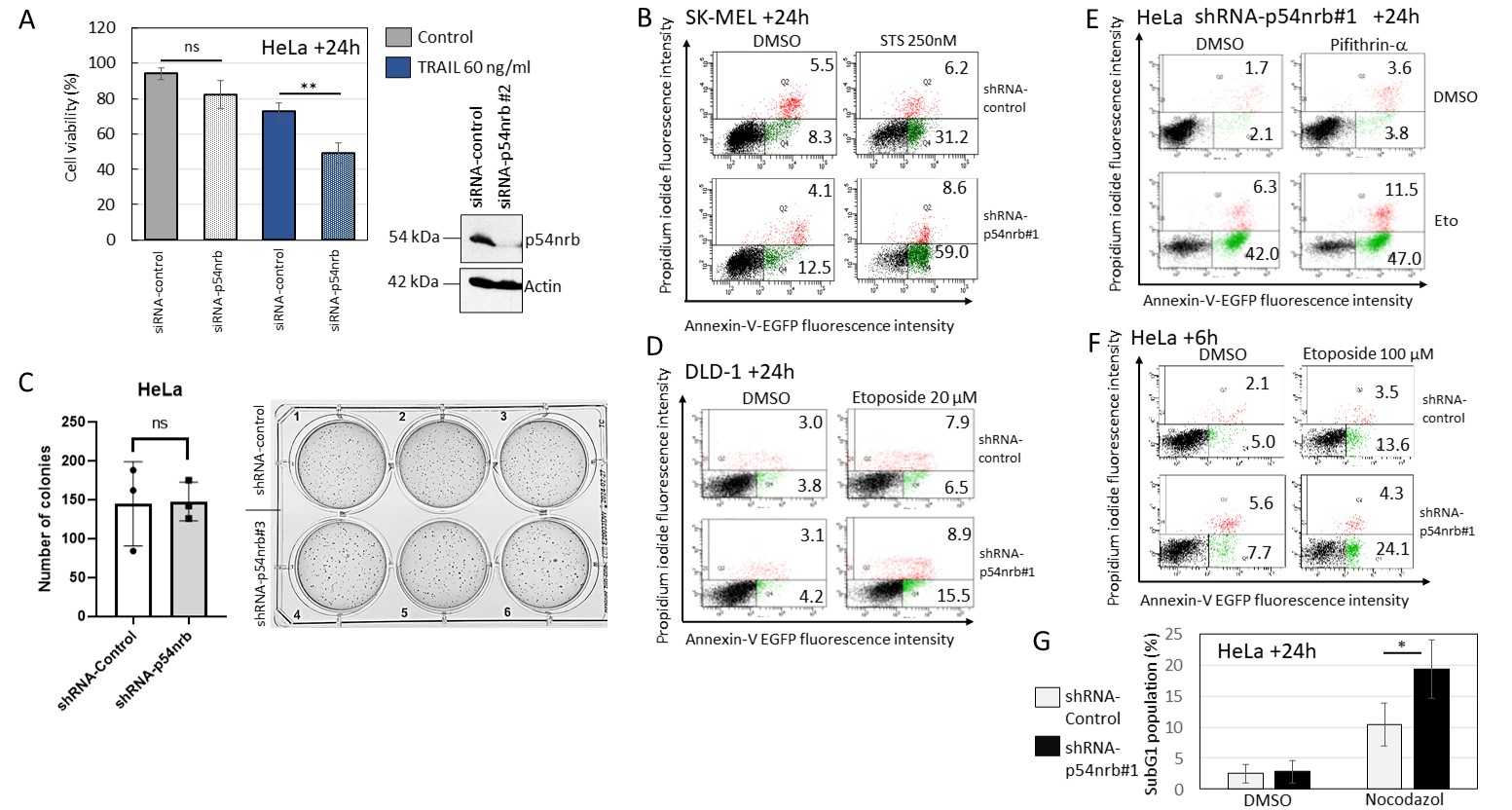

Supplement: Supplementary file 2 — Suppl Fig1 [file 41419_2022_4829_MOESM2_ESM.jpg]

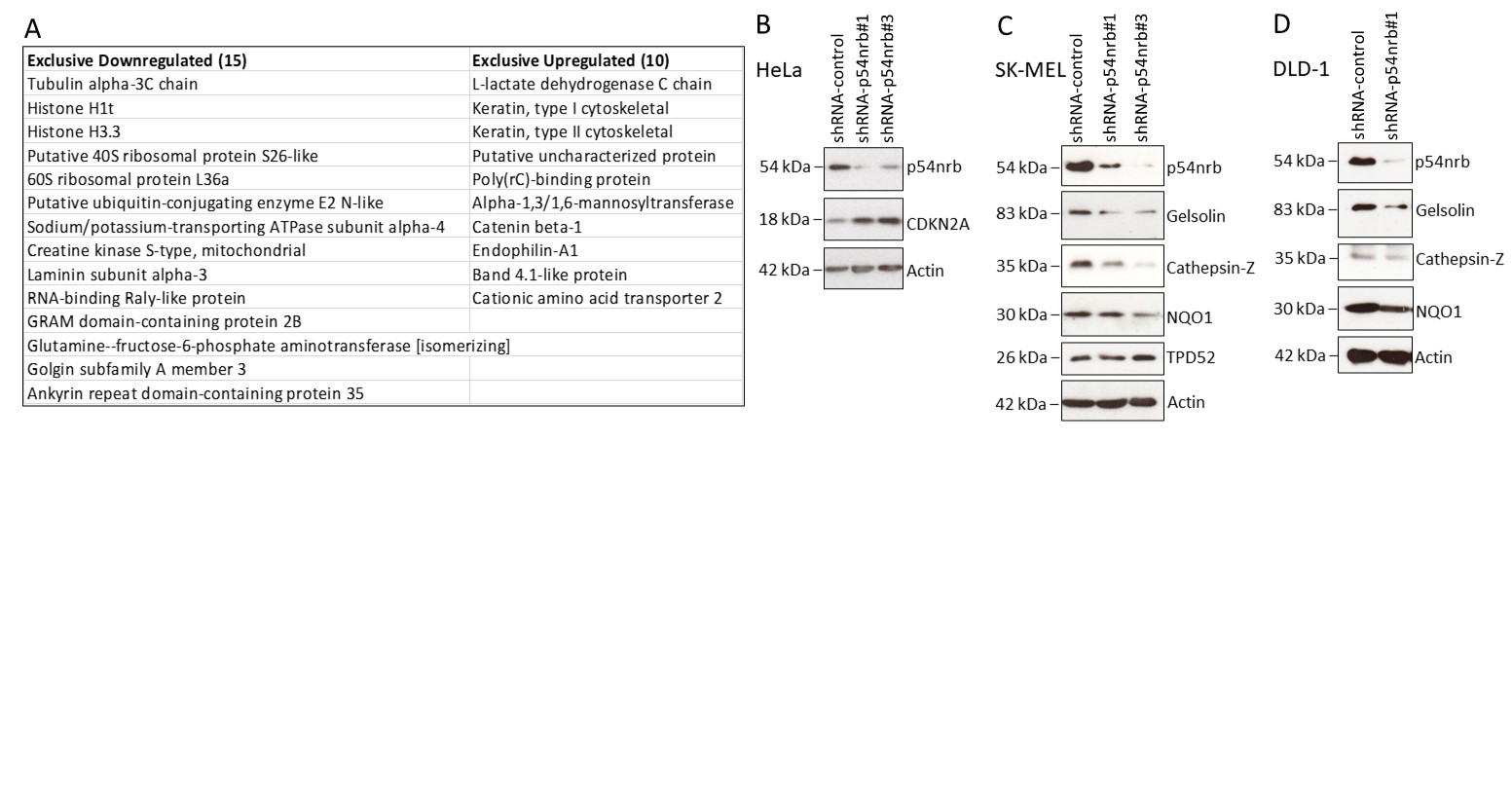

Supplement: Supplementary file 3 — Suppl Fig2 [file 41419_2022_4829_MOESM3_ESM.jpg]

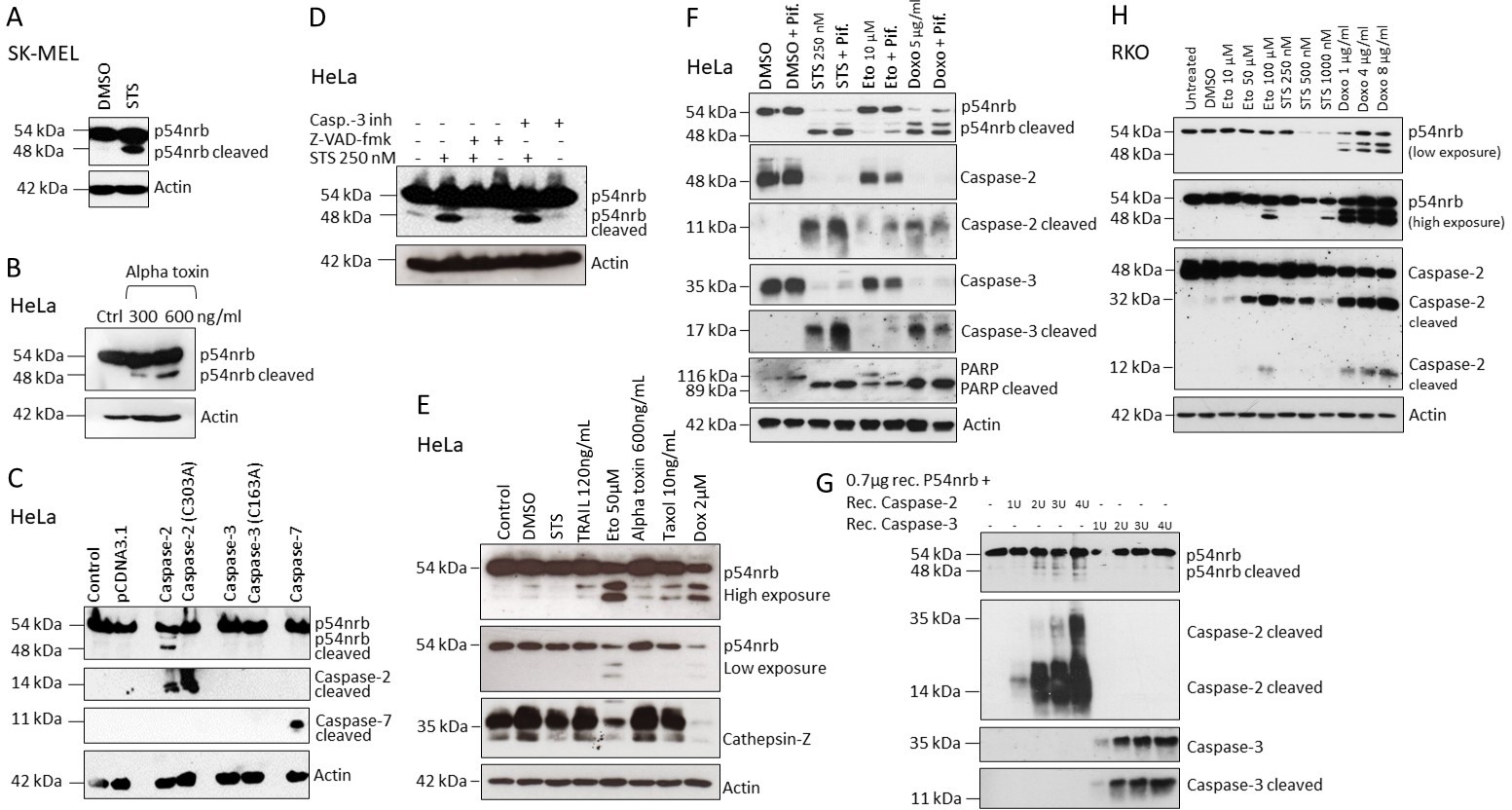

Supplement: Supplementary file 4 — Suppl Fig3 [file 41419_2022_4829_MOESM4_ESM.jpg]

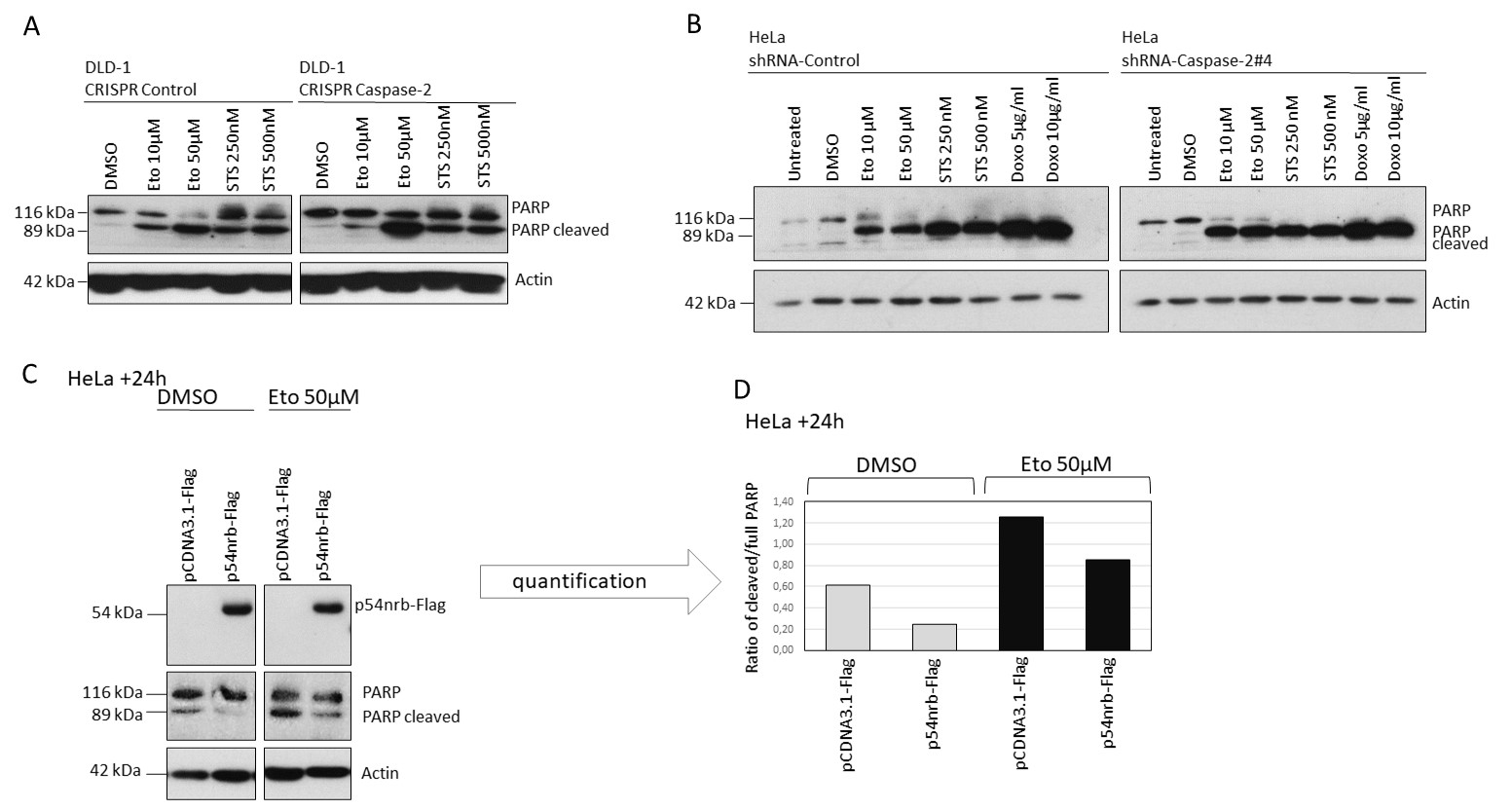

Supplement: Supplementary file 5 — Suppl Fig4 [file 41419_2022_4829_MOESM5_ESM.jpg]

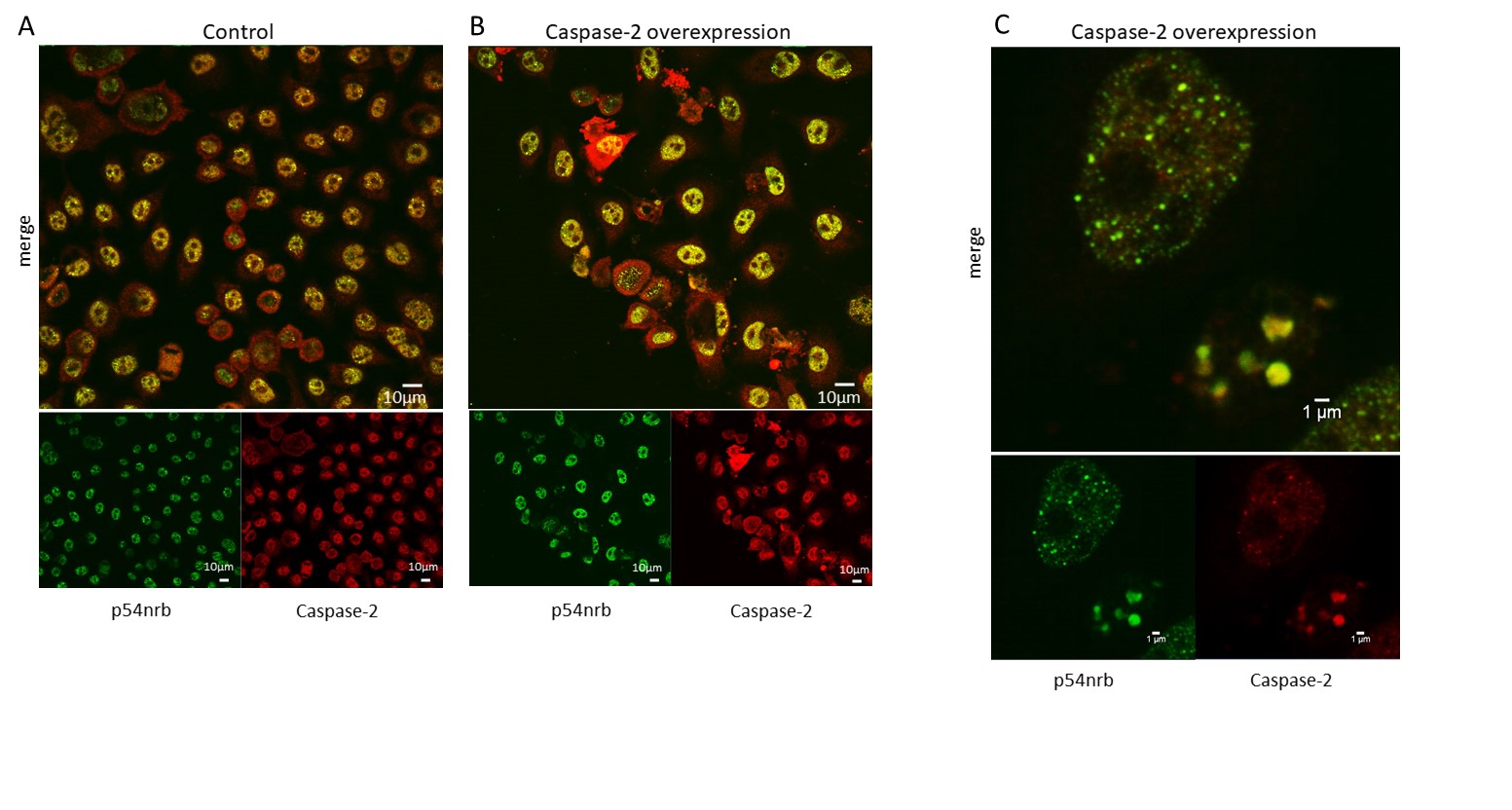

Supplement: Supplementary file 6 — Suppl Fig5 [file 41419_2022_4829_MOESM6_ESM.jpg]
