# Supplementary material for: The caspase-2 substrate p54nrb exhibits a multifaceted role in tumor cell death susceptibility via gene regulatory functions
Source: Cell Death Dis. 2022 Apr 20;13(4):386. doi: 10.1038/s41419-022-04829-2 (PMC9021192; doi:10.1038/s41419-022-04829-2)
Supplement: Supplementary file 7 — Supplementary Fig legends [file 41419_2022_4829_MOESM7_ESM.docx]

**Supplementary figure legends**

**Supplementary Fig. 1. Alteration of tumor cell survival capacity upon depletion or cleavage of p54nrb.**

(A) Left panel shows the quantification of flow cytometry detection of HeLa siRNA-p54nrb versus siRNA-control cells treated with TRAIL. Hela cells were transfected with siRNA-control or siRNA-p54nrb#2 by employing Hiperfect, and at 24 h after transfection, were treated with TRAIL. Student´s t test was employed to test significance. Ns-non significant, **p<0.01, n=3. Right panel shows the Immunoblot of p54nrb on HeLa siRNA-p54nrb versus siRNA-control cells.

(B) Flow cytometry of SK-MEL shRNA-control and shRNA-p54nrb#1 cells at 24 h after treatment with DMSO or 250nM STS. Percentage of Annexin-V-positive (green) and propidium-iodide-Annexin-V double positive (red) cells are indicated.

(C) Counts of colonies (left) and picture of one representative experiment (right) of 3D soft agar tumor growth assay from HeLa shRNA-control and shRNA-p54nrb#3 cells. 1000 cells/well were seeded and grown for 3 weeks and stained with 0.1 % crystal-violet. Student´s t test was employed to test significance. Ns-non significant, n=3. Three technical replicates were used per experiment.

(D) Flow cytometry of DLD-1 shRNA-control and shRNA-p54nrb#1 cells at 24 h after treatment with DMSO or Etoposide 20 µM. Percentage of Annexin-V-positive (green) and propidium iodide/Annexin-V double positive (red) cells are indicated.

(E) Flow cytometry of HeLa shRNA-p54nrb#1 cells. After 1 h incubation with 30 µM Pifithrin-α, the cells were treated for 24 h with DMSO or 10 µM Eto. Percentage of Annexin-V-positive (green) and propidium-iodide-Annexin-V double positive (red) cells are indicated.

(F) Flow cytometry of HeLa shRNA-control and shRNA-p54nrb#1 cells at 6 h after treatment with DMSO or 100 µM Eto. Percentage of Annexin-V-positive (green) and propidium-iodide-Annexin-V double positive (red) cells are indicated.

(G) Flow cytometry of HeLa shRNA-control (grey column) and shRNA-p54nrb#1 (black column) cells at 24 h after treatment with DMSO or 1 µg/ml Nocodazol. Percentage of SubG1 (lower DNA content due to apoptotic DNA fragmentation) population of cells are indicated.

**Supplementary Fig. 2. Gene regulatory function of p54nrb.**

(A) List of exclusive up/down-regulated proteins in LC/MS analysis (p<0.01).

(B) Immunoblot of p54nrb and CDKN2A from HeLa shRNA-control and shRNA-p54nrb cells.

(C) Immunoblot of p54nrb, gelsolin, cathepsin-Z, NQO1, and TPD52 from SK-MEL shRNA-control and shRNA-p54nrb#1 cells.

(D) Immunoblot of p54nrb, gelsolin, cathepsin-Z, and NQO1 from DLD-1 shRNA-control and shRNA-p54nrb cells.

**Supplementary Fig. 3. P54nrb cleavage in response to apoptosis stimulus or caspase-2 overexpression.**

(A) Immunoblot of p54nrb cleavage in SK-MEL cells at 24 h after treatment with 250 nM Staurosporine (STS).

(B) Immunoblot of p54nrb cleavage in HeLa cells at 24 h after treatment with 300 and 600 ng/ml alpha toxin.

(C) Immunoblot of p54nrb cleavage in HeLa cells at 48 h after ectopic expression of 1 µg/ml pcDNA3-Flag, pcDNA3-caspase-2-Flag, pcDNA3-caspase-2-C303A-Flag, pcDNA3-caspase-3-myc, pcDNA3-caspase-3-C163A-myc, and pcDNA3-caspase-7-Flag.

(D) Immunoblot of p54nrb cleavage in HeLa cells treated with a general caspase inhibitor (50 µM Z-VAD-fmk) or a non-peptide caspase-3 inhibitor (25 µM AQZ1) 1 h prior to 24 h treatment with 250 nM staurosporine (STS).

(E) Immunoblot of p54nrb cleavage and cathepsin-Z level in HeLa cells at 24 h after treatment with DMSO, 500 nM STS, 120 ng/ml TRAIL, 50 µM Eto, 600 ng/ml alpha toxin, 10 ng/ml taxol, and 2 µM doxorubicin (Dox).

(F) Immunoblot of HeLa cells treated with 30 µM Pifithrin-α (-1 h) and treated with DMSO, 250 nM STS, 10 µM Eto or 5 µg/ml Dox. Detection of p54nrb, caspase-2, caspase-3, and PARP cleavage.

(G) Immunoblot of the in vitro cleavage assay. 0.7 µg recombinant p54nrb were incubated with 1, 2, 3, or 4 U of recombinant caspase-2 or caspase-3 for 6 h. Detection of p54nrb cleavage and caspase-2 and caspase-3.

(H) Immunoblot of RKO cells treated with DMSO, Eto (10, 50, and 100 µM), STS (250, 500, and 1000 nM), or Dox (1, 4, and 8 µg/ml) for 24 h. Detection of p54nrb and caspase-2 cleavage.

**Supplementary Fig. 4**

(A) Immunoblot of PARP cleavage from DLD-1 CRISPR-control and CRISPR-caspase-2 cells, treated with DMSO, 10 and 50 µM Eto, or 250 and 500 nM STS for 24 h.

(B) Immunoblot of PARP cleavage from HeLa shRNA-control and shRNA-caspase-2#4 cells, treated with DMSO, 10 and 50 µM Eto, 250 and 500 nM STS, or 5 and 10 µg/ml Dox for 24 h.

(C) Immunoblot of p54nrb expression and PARP cleavage from HeLa shRNA-p54nrb#1 cells, transfected with either pCDNA3.1-EV-Flag or pCDNA3.1-p54nrb-Flag, then treated with DMSO or 50 µM Eto at 24 h after transfection and incubated for additional 24h.

(D) Quantification of PARP cleavage ratio in relation to the full length from Supplementary Fig. 4C with ImageJ.

**Supplementary Fig. 5**

Fluorescence microscopy of HeLa cells without (A) and with 1 µg/ml caspase-2 (pCDNA3.1-Caspae-2-Flag) overexpression for 24 h (B), stained with 2 ng/µl Anti-p54nrb-Alexa-488 (green) and and Anti-caspase-2-Alexa-647 (red). 10 µm scale is indicated. (C) Single cell close-up from caspase-2 overexpressing cells. 1 µm scale is indicated.
